# Awareness and management of cognitive impairment associated with schizophrenia in psychiatrists and patients: Results from a cross-sectional survey

**DOI:** 10.1016/j.scog.2025.100375

**Published:** 2025-06-27

**Authors:** Tomiki Sumiyoshi, Satoru Ikezawa, Kaori Inaba, Tatsuro Marumoto, Ichiro Kusumi, Kazuyuki Nakagome

**Affiliations:** a Department of Preventive Intervention for Psychiatric Disorders, National Institute of Mental Health, National Center of Neurology and Psychiatry, 4-1-1 Ogawa-Higashi, Kodaira, Tokyo 187-8551, Japan; b Nippon Boehringer Ingelheim Co., Ltd, ThinkPark Tower 2-1-1 Osaki, Shinagawa, Tokyo 141-6017, Japan; c Tomakomai Midorigaoka Hospital, 1-5-7 Shimizucho, Tomakomai, Hokkaido 053-0034, Japan; d National Center of Neurology and Psychiatry, 4-1-1 Ogawa-Higashi, Kodaira, Tokyo 187-8551, Japan

**Keywords:** Assessment, Burden of disease, Cognition, Cognitive impairment, Disease management, Japan, Schizophrenia

## Abstract

This study evaluated awareness, management, and the burden of cognitive impairment associated with schizophrenia (CIAS) in Japan. A non-interventional, cross-sectional study was conducted online between April and December 2023, involving 149 psychiatrists and 852 patients. Psychiatrists prioritized controlling positive symptoms in the acute phase of the illness, while improving social functioning was the top priority for the maintenance/stable phase. Management of CIAS was regarded as most important for the reintegration of patients into society. Psychiatrists reported higher occurrence of CIAS among inpatients than outpatients. While 72 % of psychiatrists assessed CIAS, only 15 % used the Brief Assessment of Cognition in Schizophrenia. Further, 58 % of them reported that ≤40 % of their patients received interventions for CIAS. Sixty-eight percent of patients reported current or previous experiences of CIAS. The most common CIAS-related burdens were “unable to perform tasks I could do before or they take longer” (65 %) and “unable to maintain concentration” (64 %). In patients not currently experiencing CIAS (*n* = 496), these burdens were reported by 52 % and 50 %, respectively. Although CIAS was generally recognized by psychiatrists, the use of appropriate assessment tools and interventions was not common. While many patients reported CIAS-related burdens, a substantial proportion of them were unaware of CIAS. These observations indicate that the greater awareness of CIAS may facilitate its management in clinical practice, thus enhancing the ability of patients to reintegrate into society.

## Highlights

• Psychiatrists deem cognitive symptoms a priority for patients' social reintegration.
• Use of appropriate cognitive assessments in clinical settings is limited.
• Most patients reported significant burdens related to cognitive symptoms.
• A substantial portion of patients were unaware of their cognitive impairment.

## 1 Introduction

Schizophrenia is a serious illness which causes significant socioeconomic burdens (Charlson et al., 2018; Kotzeva et al., 2023; Sado et al., 2013). It is characterized by positive symptoms (e.g., hallucination, delusions), negative symptoms (e.g., those affecting motivation, interest, or expressive functions), and impairment of several domains of cognitive function (e.g., memory, executive function, attention, processing speed, or verbal fluency) (American Psychiatric Association, 2013). Specifically, cognitive impairment associated with schizophrenia (CIAS) is highly prevalent among people with schizophrenia, with approximately 80 % of them being affected (Fujino et al., 2017; Correll and Schooler, 2020), and evidence shows that CIAS is often present during the premorbid phase of the illness (Keefe, 2014; Harvey et al., 2022).

While CIAS is associated with poor functional outcomes (Kharawala et al., 2022; Shimada et al., 2022; Sumiyoshi et al., 2016; Tominaga et al., 2018) and evidence-based treatments for CIAS are recommended in international guidelines (Vita et al., 2022), the assessment of CIAS using appropriate instruments is not prevalent in routine clinical practice (Belgaied et al., 2014). This may be because: 1) CIAS is not included in the current Diagnostic and Statistical Manual of Mental Disorders (American Psychiatric Association, 2013) (DSM) or the International Statistical Classification of Disease and Related Health Problems (World Health Organization, 2019; World Health Organization, 1990); 2) valid and feasible tools to assess CIAS are not currently available (Mascio et al., 2021), and/or 3) there are currently no approved treatments for CIAS (Javitt, 2023; Keepers et al., 2020; McCutcheon et al., 2023). Despite this, the importance of CIAS treatment has been supported by observations that patients regard the treatment of affective and cognitive symptoms to be more important than that of positive symptoms (Moritz et al., 2017), and that cognitive training can alleviate CIAS and improve social functioning (Wykes et al., 2011; Vita et al., 2021).

In spite of a gap between patients and psychiatrists regarding the needs and preferences for psychosocial treatments (Takahashi et al., 2020), to our knowledge, no large-scale studies have assessed awareness, management, and burden of CIAS in both psychiatrist and patient groups. As such, it remains unclear whether psychiatrists' awareness of CIAS affects their style of disease management (e.g., treatment selection/priorities), or whether patients' awareness of CIAS has an impact on CIAS-related burdens. To address these issues, the present survey evaluated the awareness of CIAS in both psychiatrists and patients, how CIAS is managed in clinical practice, and what burdens it places on patients. For the first time, we explored the potential impact of psychiatrist–patient interactions on the awareness of CIAS. We hypothesized that, while CIAS adversely affects daily living skills, both psychiatrists and patients do not sufficiently recognize it, and that a greater awareness of CIAS may improve its management in clinical practice.

## 2 Methods

### 2.1 Study design

This was a non-interventional, cross-sectional survey which used online questionnaires distributed from April to December 2023. This study consisted of 2 cohorts targeting psychiatrists (psychiatrist cohort) and their patients (patient cohort).

### 2.2 Ethics/approvals

This study was conducted in compliance with Guidelines for Good Pharmacoepidemiology Practice issued by the International Society for Pharmacoepidemiology, the relevant Boehringer Ingelheim Standard Operating Procedures, the Declaration of Helsinki and the Ethical Guidelines for Medical and Health Research Involving Human Subjects. This study received approval from the Institutional Review Board/Independent Ethics Committee of the Medical Corporation Toukeikai Kitamachi Clinic on April 19, 2023 (code: QTN09427).

### 2.3 Study population

Eligible psychiatrists were those working in Japan, who provided informed consent, had routinely treated ≥10 patients with schizophrenia in the prior 3 months, and could enroll ≥3 patients whose daily treatment they were responsible for. Eligible patients were those diagnosed with schizophrenia using DSM-5 or other schizophrenia diagnosis criteria for ≥1 month, were judged by their psychiatrist to be eligible to participate in the survey (e.g., able to complete the entire online questionnaire using a digital device), provided informed consent, and were aged ≥18 years at the time of consent. Patients were excluded if they had comorbid conditions, including intellectual disability, alcohol/drug abuse, epilepsy, head trauma, and/or cerebrovascular diseases.

### 2.4 Recruitment

This study aimed to enroll 150 psychiatrists and ≤1000 patients. Site selection was conducted between February and September 2023. For the primary recruitment method, candidate sites, including hospitals and clinics, were randomly selected from the database owned by IQVIA Solutions Japan K.K., which contains comprehensive nationwide pharmaceutical sales data provided by pharmaceutical wholesalers in Japan. The top 3 most commonly prescribed typical and atypical antipsychotics were identified, and sites with confirmed prescriptions of these medications were randomly selected to ensure generalizability of the results. The secondary recruitment method, used to reach the planned sample size, allowed for sites which had agreed to participate in the study and site management organizations to introduce other facilities as additional candidate sites.

To ensure data were captured across different types of institutions, the intended proportion of site types was 30 % university hospitals, 35 % general hospitals, and 35 % clinics. The online questionnaire was sent to up to 5 psychiatrists who met the eligibility criteria per site. Various types of psychiatrists were invited based on their specialties, positions, and number of patients. The patients were introduced consecutively by psychiatrists who were participating in the survey, and the online questionnaire was sent to eligible patients. To control for selection bias, the number of patients introduced by a single psychiatrist was limited up to 12.

### 2.5 Data sources

All data were obtained through a self-administered online survey conducted using the Qualtrics platform (Qualtrics LLC, Provo UT, USA). The questionnaires included structured multiple answer and yes/no questions (Supplementary Appendix). The survey was conducted once for each respondent; if multiple responses were obtained with the same identification/login, only the results of the most recent response were used in the analysis. All survey data were stored on the Qualtrics server, and were encrypted, secured, and made accessible only by authorized personnel. All personal information, including names and contact details, was anonymized, and stored in the study sites, but not on the Qualtrics platform.

### 2.6 Analysis

The primary objective was to clarify the awareness of CIAS in psychiatrists and patients, and how CIAS is managed in daily clinical practice. The survey for psychiatrists targeted recognition of CIAS as a treatment goal, perception of CIAS in clinical practice, percentage of patients presenting with CIAS, history of CIAS assessment in clinical practice, percentage of patients receiving treatment for CIAS, and types of treatment for CIAS, while the survey for patients concerned awareness of experiencing CIAS.

The second primary objective was to identify CIAS-related burdens perceived by patients, patient needs, and motivation for the treatment of CIAS, while the survey for patients assessed perceived burdens and unmet needs related to CIAS.

The secondary objective was to determine whether patient–psychiatrist interactions affect the awareness of CIAS by patients. This was evaluated by assessing 1) the awareness of CIAS by psychiatrists and the explanations of it to patients, and 2) the awareness of CIAS by patients and their recognition that their psychiatrists had explained CIAS to them.

Data were analyzed descriptively. Continuous data were described by the number of non-missing values, mean and standard deviation (SD). Frequency and percentages were reported for categorical data.

## 3 Results

### 3.1 Study populations

Overall, 67 sites were recruited (34 via the primary recruitment method and 33 via the secondary recruitment method; Supplementary Fig. 1). Of those, 19 (28 %) were university hospitals (departments of psychiatry), 21 (31 %) were general hospitals (departments of psychiatry) or single-department psychiatric hospitals, and 27 (40 %) were psychiatric clinics. Of the 151 psychiatrists who completed the survey, 149 were included in the analysis; two of them were excluded for providing multiple responses (*n* = 1) and completing the questionnaire in an unrealistically short time frame (n = 1). Of the 869 patients who completed the survey, 852 were included in the analysis; reasons for exclusion were providing multiple responses (*n* = 15) and being an unidentified patient or giving information on an unidentified institution (*n* = 2).

### 3.2 Demographics and clinical characteristics

Most psychiatrists (*n* = 98, 66 %) had treated >30 patients with schizophrenia in the prior 3 months, and attended ≥1 academic conference annually (*n* = 120, 81 %) (Table 1). Psychiatrists reported that, on average, 26 % of their patients were in the acute phase and 74 % were in the maintenance/stable phase of schizophrenia. In the patient cohort, 99 % (*n* = 840) reported that they were currently receiving pharmacological treatment, while 19 % (*n* = 161) reported receiving a non-pharmacological treatment (Table 2). Around two-thirds of patients (*n* = 582, 68 %) reported experiencing or having previously experienced CIAS. The use of non-pharmacological treatments among patients currently experiencing CIAS was rare, with only a small percentage receiving interventions, such as social skills training, cognitive behavioral therapy (CBT), and cognitive rehabilitation.

**Table 1:** Demographics of the psychiatrist cohort.

			Degree of awareness of CIAS^a^		
		Overall (*n* = 149)	Group 1 (*n* = 21)	Group 2 (*n* = 73)	Group 3 (*n* = 55)
Age, years, n (%)	≤39	52 (35)	8 (38)	26 (36)	18 (33)
	40–59	75 (50)	10 (48)	38 (52)	27 (49)
	≥60	22 (15)	3 (14)	9 (12)	10 (18)
Institution type, n (%)	Psychiatric hospitals	45 (30)	7 (33)	28 (38)	10 (18)
	Department of psychiatry at general hospitals	22 (15)	4 (19)	6 (8)	12 (22)
	Department of psychiatry or psychosomatic medicine at academic medical centers	53 (36)	7 (33)	22 (30)	24 (44)
	Psychiatric or psychosomatic medicine clinics	29 (19)	3 (14)	17 (23)	9 (16)
Research/learning experience related to schizophrenia, n (%)	Clinical studies	81 (54)	9 (43)	39 (53)	33 (60)
	Sponsor- or investigator-initiated clinical trials	64 (43)	7 (33)	30 (41)	27 (49)
	Academic conferences (≥1 per year)	120 (81)	15 (71)	62 (85)	43 (78)
	Local seminars (≥1 per year)	92 (62)	11 (52)	45 (62)	36 (65)
Number of patients with schizophrenia they treated in the preceding 3 months, n (%)	≥100	26 (17)	1 (5)	15 (21)	10 (18)
	61–99	24 (16)	5 (24)	10 (14)	9 (16)
	31–60	48 (32)	6 (29)	25 (34)	17 (31)
	1–30	51 (34)	9 (43)	23 (32)	19 (35)
Disease stage of patients with schizophrenia, %, mean (standard deviation)	Acute phase	26 (22)	35 (25)	22 (20)	28 (21)
	Maintenance/stable phase	74 (22)	65 (25)	78 (20)	72 (21)

a Psychiatrists were categorized into 3 groups based on the number of correct answers to questions about CIAS (out of 16 points; Group 1, 9–12; Group 2, 13–14; and Group 3, 15–16).

**Table 2:** Demographics of the patient cohort.

			CIAS experience		
		Overall (*n* = 852)	Currently experiencing (*n* = 356)	Previously experienced (*n* = 226)	Never experienced (*n* = 270)
Age, years, n (%)	≤29	94 (11)	43 (12)	31 (14)	20 (7)
	30–49	398 (47)	169 (47)	117 (52)	112 (41)
	≥50	360 (42)	144 (40)	78 (35)	138 (51)
Gender, n (%)	Female	380 (45)	149 (42)	96 (42)	135 (50)
	Male	465 (55)	202 (57)	128 (57)	135 (50)
	Neither/do not want to answer	7 (1)	5 (1)	2 (1)	0 (0)
Employed, n (%)	Yes	527 (62)	207 (58)	159 (70)	161 (60)
	Full-time employee (as regular worker)	38 (7)	8 (4)	17 (11)	13 (8)
	Full-time employee (as person with disability)	22 (4)	10 (5)	9 (6)	3 (2)
	Temporary employee	35 (7)	14 (7)	12 (8)	9 (6)
	Self-employed	34 (6)	9 (4)	9 (6)	16 (10)
	Part-time employee	106 (20)	32 (15)	37 (23)	37 (23)
	Type A workplace^a^	36 (7)	18 (9)	11 (7)	7 (4)
	Type B workplace^a^	107 (20)	51 (25)	22 (14)	34 (21)
	Employment transition support	16 (3)	7 (3)	6 (4)	3 (2)
	Housemaker	111 (21)	47 (23)	31 (19)	33 (20)
	Student	18 (3)	10 (5)	5 (3)	3 (2)
	Other	4 (1)	1 (0.5)	0	3 (2)
Current pharmacological treatment for schizophrenia, n (%)	Currently receiving	840 (99)	351 (99)	224 (99)	265 (98)
	Previously received	3 (0.4)	2 (1)	0 (0)	1 (0.4)
	Have never received	7 (1)	3 (1)	2 (1)	2 (1)
	Do not know	2 (0.2)	0 (0)	0 (0)	2 (1)
Status of non-pharmacological treatment for schizophrenia, n (%)	Currently receiving	161 (19)	89 (25)	40 (18)	32 (12)
	Never received	579 (68)	220 (62)	158 (70)	201 (74)
	Do not know	112 (13)	47 (13)	28 (12)	37 (14)
Current non-pharmacological therapy for schizophrenia, n (%)	Cognitive rehabilitation	25 (3)	9 (3)	10 (4)	6 (2)
	Social skills training	92 (11)	53 (15)	23 (10)	16 (6)
	Cognitive behavioral therapy	45 (5)	26 (7)	15 (7)	4 (1)
	Transcranial magnetic stimulation	9 (1)	7 (2)	2 (1)	0 (0)
	Electroconvulsive therapy	47 (6)	26 (7)	11 (5)	10 (4)

a Type A workplace refers to continued employment support with a salary/contract, Type B workplace refers to continued employment support with no contract but paid wages.

### 3.3 Treatment priorities for psychiatrists

**Fig. 1:**
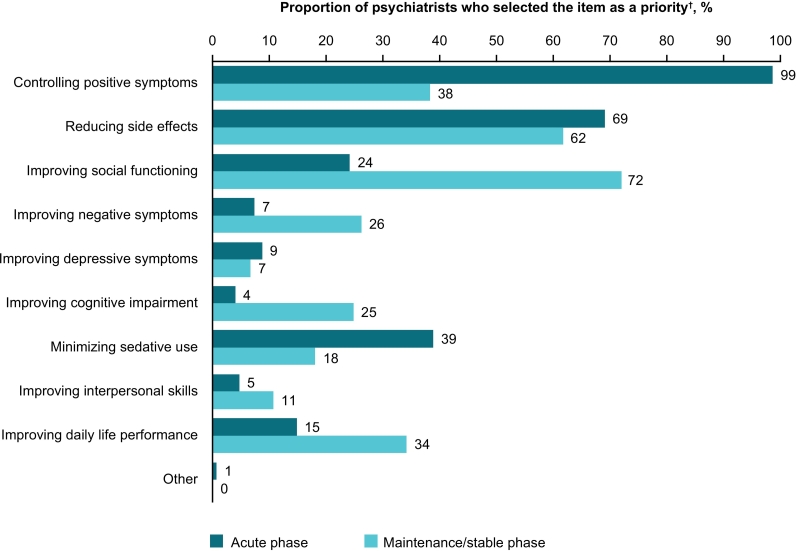
Psychiatrists' opinion on treatment priorities in the acute phase and maintenance/stable phase of schizophrenia. ^†^Psychiatrists could select up to 3 items.

The top priorities for psychiatrists in the treatment of the acute phase of schizophrenia were controlling positive symptoms (*n* = 147, 99 %) and reducing side effects (*n* = 103, 69 %), while improving cognitive impairment was reported as a priority by only 6 (4 %) psychiatrists (Fig. 1). The top priorities in the treatment of the maintenance/stable phase were improving social functioning (*n* = 107, 72 %) and reducing side effects (*n* = 92, 62 %); improving cognitive impairment was reported as a priority by 37 (25 %) psychiatrists.

**Fig. 2:**
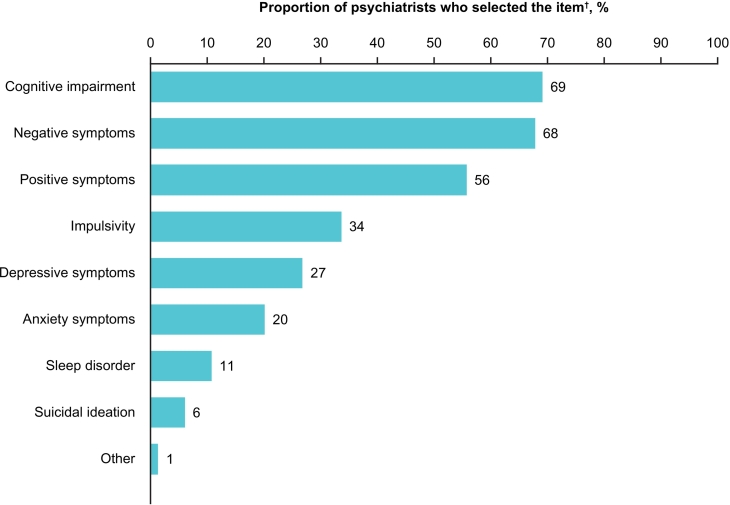
Psychiatrists' opinion on treatment priority for the reintegration of patients into society. ^†^Psychiatrists could select up to 3 items.

When asked which clinical symptoms are particularly important to treat for the reintegration of patients into society, cognitive impairment, negative symptoms and positive symptoms were nominated by 69 % (*n* = 103), 68 % (*n* = 101) and 56 % (*n* = 83) of psychiatrists, respectively (Fig. 2).

### 3.4 Awareness of CIAS by psychiatrists

**Fig. 3:**
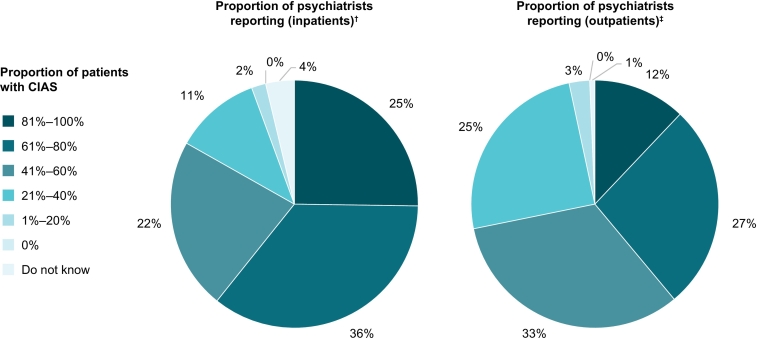
Proportion of inpatients and outpatients with CIAS as reported by psychiatrists. ^†^Psychiatrists who had treated inpatients in the past 3 months (*n* = 107). ^‡^All psychiatrists (n = 149).

Psychiatrists reported higher occurrence of CIAS among inpatients than outpatients (Fig. 3). The proportion of psychiatrists reporting that >80 % of their patients had CIAS was more than twice as high for inpatients (25 % [*n* = 27/107]) compared with outpatients (12 % [*n* = 18/149]).

The level of understanding of CIAS by psychiatrists was assessed with 16 questions, yielding the mean (SD) score of 13.9 (1.4) out of 16 (Supplementary Fig. 2). The majority of psychiatrists (*n* = 92, 62 %) correctly identified that CIAS presents before the onset of the illness, and 75 (50 %) correctly answered that CBT is not a type of cognitive rehabilitation. Psychiatrists with a better understanding of CIAS reported a higher proportion of their patients live with it in both inpatient and outpatient settings (Supplementary Fig. 3).

### 3.5 Assessment and management of CIAS in clinical practice

Overall, 108 psychiatrists (72 %) responded that they evaluated CIAS in clinical practice, of whom 94 (87 %) used interview-based assessments, 52 (48 %) used test-based assessments, and 1 (1 %) reported using other assessment types. Thirty seven (25 %) psychiatrists used objective tests, i.e., the Brief Assessment of Cognition in Schizophrenia (BACS), Cognitive Assessment Interview (CAI), Digit Symbol Substitution Test (DSST), Japanese Adult Reading Test (JART), MATRICS Consensus Cognitive Battery (MCCB), Repeatable Battery for the Assessment of Neuropsychological Status (RBANS), Schizophrenia Cognition Rating Scale (SCoRS), and/or Wechsler Adult Intelligence Scale 3rd Edition (WAIS-III). Among the 52 psychiatrists who used test-based assessments, the most common methods were Brain Imaging Test (BIT), Global Assessment of Functioning (GAF), Hasegawa Dementia Scale (HDS), and Mini Mental State Examination (MMSE) (Supplementary Fig. 4). None of the 149 psychiatrists implemented MCCB, and only 22 (15 %) implemented BACS.

**Fig. 4:**
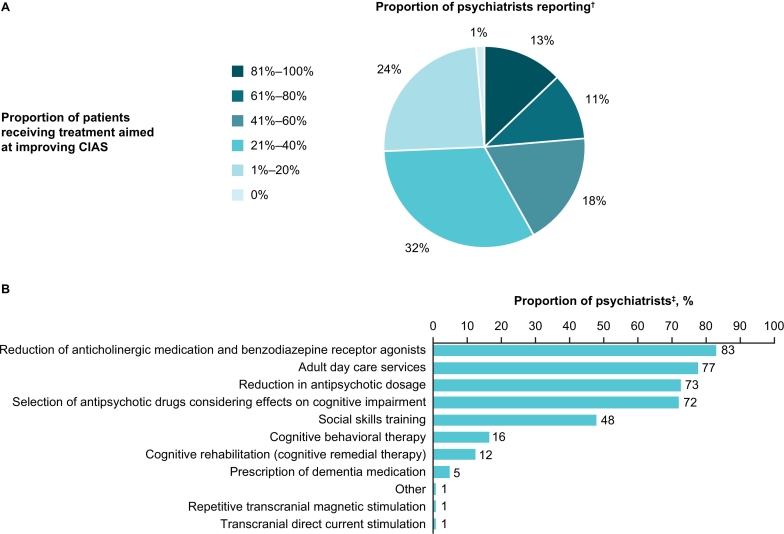
Interventions for CIAS in clinical practice as reported by psychiatrists. (A) Proportion of patients receiving interventions aimed at improving CIAS^†^ and (B) type of interventions for CIAS^‡^. ^†^Psychiatrists who answered that they provide treatment for CIAS in either inpatient or outpatient settings (*n* = 148). ^‡^Psychiatrists who responded that patients were receiving treatment aimed at improving CIAS (*n* = 146).

Fifty-eight percent (*n* = 86) of psychiatrists reported that ≤40 % of their patients were receiving interventions for CIAS (Fig. 4A). Most psychiatrists reported these interventions were related to reducing negative effects of medications on CIAS, and that they were providing adult day care services (Fig. 4B).

Proactivity of psychiatrists in explaining clinical symptoms to patients and caregivers were captured (Supplementary Fig. 5); 89 % (*n* = 133) of psychiatrists reported initiating explanation of positive symptoms to patients, whereas 48 % (*n* = 71) did so for CIAS.

### 3.6 Awareness of CIAS and disease burden experienced by patients

**Fig. 5:**
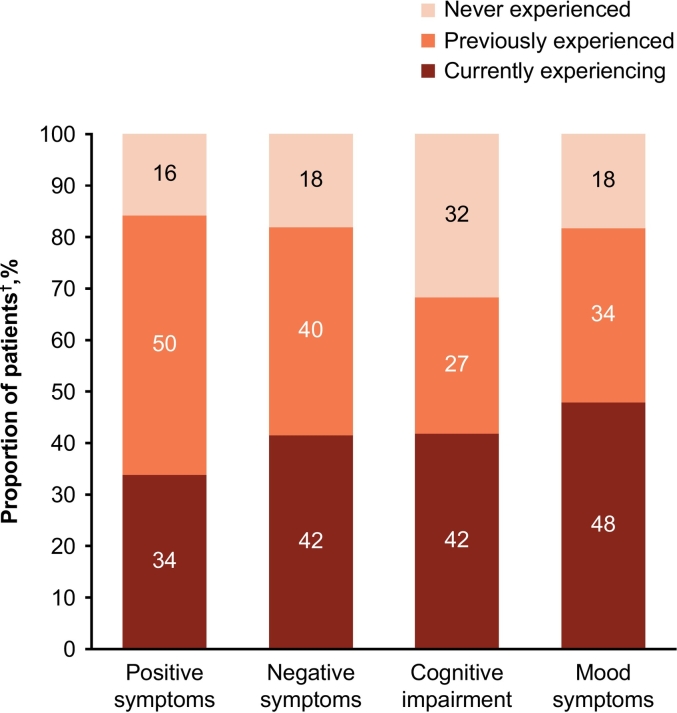
Experience of symptoms by patients. ^†^All patients (n = 852).

**Fig. 6:**
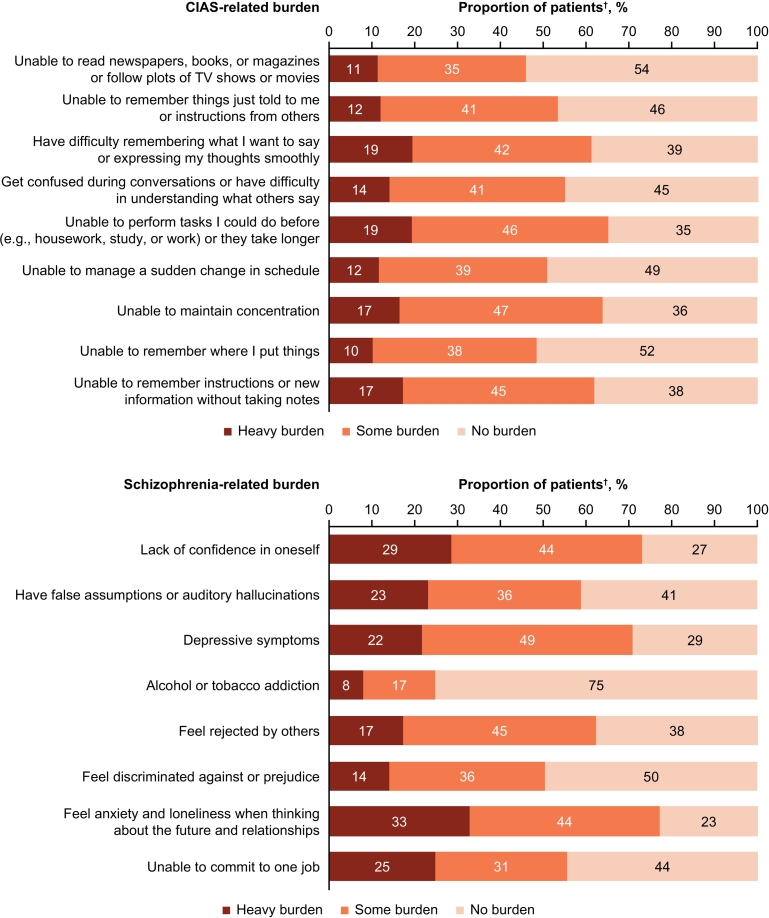
Disease burden experienced by patients. ^†^All patients (n = 852).

Overall, 68 % (*n* = 582) of patients reported they were currently experiencing or had previously experienced CIAS, while over 80 % reported currently or previously experiencing positive, negative, or mood symptoms (Fig. 5). When asked about schizophrenia and CIAS-related burdens, the highest ranked CIAS-related burdens (i.e., >60 % heavy burden/some burden) were inability or taking longer than usual to complete tasks, inability to maintain concentration, and difficulty remembering instructions or new information (Fig. 6). The most common heavy burden related to CIAS was “Have difficulty remembering what I want to say or expressing my thoughts smoothly” (*n* = 166, 19 %). Among the 496 patients who reported not currently experiencing CIAS, the related burdens were “Unable to perform tasks I could do before (e.g., housework, study, or work) or they take longer” (*n* = 260, 52 %), “Unable to maintain concentration” (*n* = 250, 50 %), and “Unable to remember instructions or new information without taking notes” (*n* = 233, 47 %).

Seventy-six percent (*n* = 632) of patients reported unmet needs with their current treatments. When asked about what they “have been unable to do but would like to be able to do in the future”, traveling was the most common answer (*n* = 182, 22 %), followed by marriage (*n* = 176, 21 %), and improvement in working conditions (*n* = 175, 21 %) (Supplementary Fig. 6).

### 3.7 Impact of psychiatrist–patient interactions on the awareness of CIAS

**Fig. 7:**
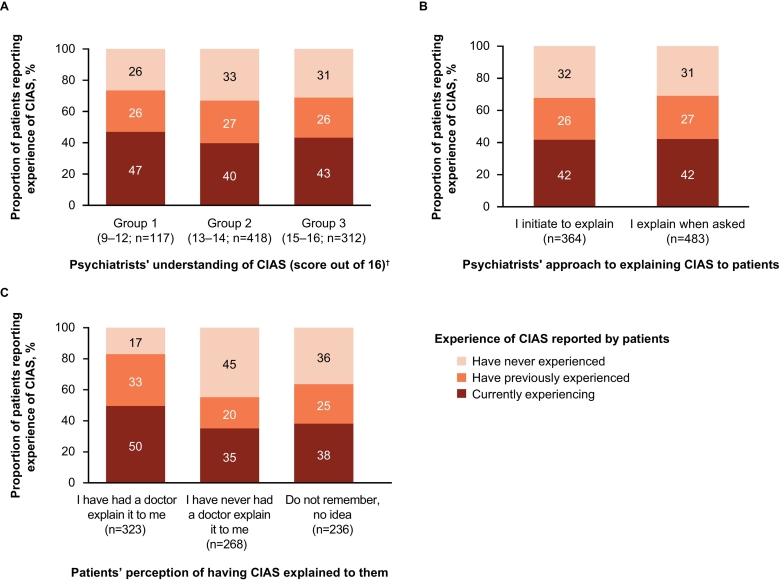
Interactions between psychiatrists and patients on the awareness of CIAS. (A) Psychiatrists' understanding of CIAS, (B) psychiatrists' proactivity with explaining CIAS, and (C) patients' experiences of having CIAS explained to them. Psychiatrist–patient relationships were analyzed for psychiatrists who enrolled ≥3 schizophrenia patients (n = 133) and the corresponding patients (*n* = 827). ^†^Psychiatrists answered 16 questions to assess their understanding of CIAS. Psychiatrists were categorized into 3 groups based on their scores out of 16: Group 1, score 9–12 (n = 21); Group 2, score 13–14 (n = 73); and Group 3, score 15–16 (n = 55).

To examine how interactions between psychiatrists and patients affect the awareness of CIAS, experiences of CIAS in patients were analyzed based on the psychiatrists' understanding of CIAS (Fig. 7A), proactivity in explaining CIAS (Fig. 7B), or patients' experiences of having had CIAS explained to them (Fig. 7C). There was no notable association between patients' experience of CIAS and psychiatrists' understanding of it or their proactivity in explaining CIAS to patients (active initiation vs. on request). However, patients who had perceived receiving an explanation of CIAS were more likely to be currently experiencing or have previously experienced CIAS than those who had never been given explanations (or did not remember if they had). Of the 364 patients treated by psychiatrists who answered “I initiate to explain” CIAS, 150 (41 %) responded that they had received an explanation about it (Supplementary Fig. 7). Among the 186 patients whose psychiatrists used materials and/or web pages provided by a pharmaceutical company to explain CIAS, 43 % reported they had received an explanation about it. The corresponding figure was 37 % in 521 patients whose psychiatrists gave them only verbal explanations (Supplementary Fig. 8).

## 4 Discussion

The present study is the first large-scale survey participated by both psychiatrists and patients, providing a comprehensive overview and insight into the awareness and practices on CIAS. Patients were referred by their psychiatrists, ensuring a more reliable inclusion of patients compared to those included in typical web-based surveys. Furthermore, analysis of psychiatrist–patient interactions allowed us to investigate the degree of CIAS awareness among patients in relation to the explanations they received from their treating psychiatrists.

The majority of psychiatrists surveyed in this study were practicing actively, and had treated >30 patients with schizophrenia in the past 3 months. Furthermore, these psychiatrists appeared to be highly motivated to learn, with most of them attending ≥1 conference annually. Of the 852 patients included in the study, most were receiving pharmacological treatments, whereas only a small proportion of them were also receiving non-pharmacological therapy, consistent with the results reported by psychiatrists. While two-thirds of patients reported current or previous experiences of CIAS, a very small proportion was receiving evidence-based interventions for CIAS (with 3 % receiving cognitive rehabilitation), suggesting an insufficient understanding of CIAS among treating psychiatrists.

Psychiatrists prioritized “improving social functioning” for treatment goals in the maintenance/stable phase, with treatment of “cognitive impairment” being the most important factor for the reintegration of patients into society. This may align with the association between CIAS and social functioning, as reported in previous studies (Kharawala et al., 2022; Sumiyoshi et al., 2016; Tominaga et al., 2018). Accordingly, another online survey with 229 psychiatrists in Japan found 86.5 % of them believed improving CIAS would lead to better social functioning (Shinohara et al., 2024). While the psychiatrists who participated in the current study regarded cognitive impairment as a high treatment priority in *stabilized* patients, this was not the case for patients with *first or acute episodes*, consistent with the results from the FOCIS international survey (Green et al., 2005).

Psychiatrists surveyed in this study regarded CIAS to be more common in inpatients than outpatients, aligning with previous observation that outpatients usually have better cognitive function than inpatients, even after adjusting for demographics and psychiatric symptoms (Kurebayashi and Otaki, 2018). A review of 30 longitudinal studies also reported that cognitive symptoms worsened over the course of schizophrenia, and were particularly poor in inpatients (Ojeda et al., 2007), while worsening of cognitive function might impede the discharge of inpatients (Shimono et al., 2004).

Responses to the questionnaires suggest a lack of sufficient understanding of CIAS among psychiatrists. For example, half of them incorrectly answered that “CBT is a type of cognitive rehabilitation”. This might be due to the low penetration of CBT in Japan (Yoshinaga et al., 2015; Hayashi et al., 2020; Kobori et al., 2014); only 38 % (*n* = 386/1019) of psychiatric clinics were found to be implementing CBT (Takahashi et al., 2018). Numerous studies have shown the presence of CIAS before the onset of schizophrenia (Keefe, 2014; Harvey et al., 2022). Despite this, 38 % of psychiatrists did not recognize that CIAS is present before the onset of schizophrenia, although they were participating in clinical research and frequently attending conferences. Therefore, specific efforts are needed to promote the understanding of CIAS among psychiatrists.

Despite the high prevalence of CIAS (Fujino et al., 2017; Correll and Schooler, 2020), the assessment of cognitive function with appropriate instruments is not common in clinical settings (Belgaied et al., 2014). In this study, no participants used the MCCB, and only 15 % used BACS, although both measures are considered gold-standard for the evaluation of CIAS (Javitt, 2023). These results align with a previous survey involving 229 Japanese psychiatrists who evaluated CIAS (Shinohara et al., 2024). In that study, they commonly used the MMSE, HDS-R, WAIS, and GAF (68.5 %, 61.1 %, 50.0 %, and 49.1 %) (Shinohara et al., 2024), while only 25.9 %, 6.5 %, and 1.9 % of them used the BACS, SCoRS, and MCCB, respectively (Shinohara et al., 2024). Assessment with MMSE or HDS-R requires only around 10 min, and are reimbursable under insurance, which may explain why psychiatrists are more aware of/more likely to use them. However, these assessment tools are better suited to evaluating cognition and intellectual functioning in dementia (Galea and Woodward, 2005; Gong et al., 2021).

More than half of the psychiatrists reported that ≤40 % of their patients were receiving interventions for CIAS. However, the intervention options are limited due to the absence of established methods (McCutcheon et al., 2023). The national pharmacological treatment guidelines for schizophrenia in Japan recommend the use of second-generation antipsychotic drugs rather than first-generation antipsychotics for patients with cognitive dysfunction, while the use of anticholinergics and benzodiazepines is discouraged (Japanese Society of Neuropsychopharmacology and Japanese Society of Clinical Neuropsychopharmacology, 2025). Therefore, the present results may reflect clinical practices attempting to follow the recommendations of a local guideline. A previous study reported the most common method to treat CIAS was pharmacotherapy (76.1 %) (Shinohara et al., 2024), consistent with our results where only a small portion of psychiatrists used cognitive rehabilitation. This may be due to the difficulty of introducing psychosocial therapies into daily practices, as the national insurance in Japan does not cover the costs for securing skilled personnel and equipment.

Cognitive domains commonly reported to be impaired in people with schizophrenia include verbal memory, processing speed, concentration, and working memory (Keefe et al., 2011). These areas of cognitive function may reflect the burdens most frequently reported by the patients surveyed in this study, e.g., inability to do housework, maintain concentration, and difficulty remembering instructions or new information. On the other hand, less than half of the psychiatrists studied here actively explained cognitive symptoms to their patients, although treating cognitive and emotional problems has been reported to be more important than treating positive symptoms, even in the active phase of schizophrenia (Moritz et al., 2017). There was a discrepancy between the occurrence of CIAS-related burdens (in around 60 % of participants) and the ongoing experience of CIAS (around 40 %). This suggests that some patients may not recognize their CIAS-related burdens as being due to CIAS or that patients are unaware of it. This assumption is supported by a study which showed more than half of individuals with schizophrenia appear unaware of CIAS that was objectively measured (Burton et al., 2016). In sum, CIAS should be more frequently discussed between psychiatrists and patients to improve the quality of treatments and long-term outcomes.

Regarding the treatment goals patients would like to achieve, “traveling” was the most common, followed by “marriage” and “improvement in working conditions”. As cognitive function has been reported to be strongly associated with independent living and occupational function (Kharawala et al., 2022), maintaining and improving cognition may help patients achieve these personal goals.

In this study, no clear association was found between the CIAS reported by patients and whether psychiatrists proactively explained it to them. This suggests that explanations of CIAS may not immediately facilitate its awareness by patients. However, it is important to note that patients who perceived that their psychiatrists had explained CIAS to them were more likely to report experiencing CIAS than those who did not perceive such explanation. CIAS is composed of multiple domains, and affects several aspects of daily life (Correll and Schooler, 2020; Keefe et al., 2011). Therefore, it is possible that patients may not fully understand CIAS depending on how it is explained to them. This is supported by the current observation that among patients treated by psychiatrists who answered, “I initiate to explain” (about CIAS), less than half responded that their psychiatrists had explained it. Further, the present results indicate the use of materials and/or web pages is more efficacious than verbal explanation alone for the enlightenment of CIAS. Therefore, relating CIAS to specific difficulties experienced in daily life, preferably with visual aids, may help promote its recognition by patients. Overall, the results of this study underscore the importance of such educational approaches to enhance the awareness of CIAS in patients.

The clinical implications of the observed findings should be mentioned. A better understanding of, and insight into, CIAS among psychiatrists may improve treatment outcomes for patients with schizophrenia. Specifically, strategies to improve psychiatrists' understanding of CIAS could include education on the prevalence and impact of CIAS, which may be facilitated by the development and dissemination of psychometrically robust and clinically feasible assessment tools. In addition, disseminating evidence-based treatments for CIAS, including psychosocial interventions such as cognitive remediation, may encourage their integration into clinical practice. By recognizing CIAS as a key factor affecting social functioning, psychiatrists can prioritize interventions that target cognitive symptoms.

There are limitations to consider when interpreting the results of this study. First, the population may not be accurately represented due to potential selection bias from the survey methodology. Second, the analysis of the relationship between psychiatrist–patient interactions and awareness of CIAS is exploratory, and was not adjusted for multiple confounding factors. Third, it was challenging in the context of a cross-sectional study to determine whether patients' awareness of CIAS is associated with their perception of having had it explained to them by their treating psychiatrists. Therefore, further research is required to examine the causality. Fourth, the generalizability of the current findings may be limited, as the data were collected within a single national healthcare context.

In conclusion, this is the first large-scale survey to investigate the awareness and management of CIAS among psychiatrists and patients. Psychiatrists surveyed in this study regarded improving social functioning as the top priority for maintaining the stable phase of schizophrenia. They also believed that addressing CIAS can most effectively facilitate reintegration of patients into society. While most psychiatrists reported the presence of CIAS in the majority of their patients, only a small proportion of them were assessed with appropriate instruments or received interventions for CIAS. Approximately two-thirds of patients experienced disturbances of cognitive domains, including processing speed, concentration and working memory. The results also indicate that a considerable proportion of patients may not be aware of CIAS. Taken together, effective education for both psychiatrists and patients may enhance the awareness of CIAS and reduce the related burdens. These efforts are expected to improve social functioning and quality of daily life for individuals with schizophrenia.

## CRediT authorship contribution statement

**Tomiki Sumiyoshi:** Writing – review & editing, Methodology, Investigation, Formal analysis, Conceptualization. **Satoru Ikezawa:** Writing – review & editing, Methodology, Investigation, Formal analysis, Conceptualization. **Kaori Inaba:** Writing – review & editing, Project administration, Methodology, Investigation, Formal analysis, Conceptualization. **Tatsuro Marumoto:** Writing – review & editing, Writing – original draft, Project administration, Formal analysis. **Ichiro Kusumi:** Writing – review & editing, Methodology, Investigation, Formal analysis, Conceptualization. **Kazuyuki Nakagome:** Writing – review & editing, Methodology, Investigation, Formal analysis, Conceptualization.

## Funding statement

This study was funded by 10.13039/100001003Boehringer Ingelheim (BI study number: 1346-0068). Boehringer Ingelheim was given the opportunity to review the manuscript for medical and scientific accuracy as well as intellectual property considerations. The authors did not receive payment related to the development of the manuscript.

## Declaration of competing interest

TS received consulting fees and honoraria from Nippon Boehringer Ingelheim Co., Ltd., Sumitomo Pharma Co., Ltd., Otsuka Pharmaceutical Co., Ltd., Takeda Pharmaceutical Co., Ltd., and Lundbeck Japan K.K.; received payment for expert testimony from Nippon Boehringer Ingelheim Co., Ltd.; received support for attending meetings from Nippon Boehringer Ingelheim Co., Ltd.; and is a member of the Nippon Boehringer Ingelheim Co., Ltd. Advisory Board.

SI has received speaker honoraria from Boehringer Ingelheim Pharmaceuticals, Lundbeck, Sumitomo Pharma, and Takeda Pharmaceuticals, and consulting fees from Boehringer Ingelheim Pharmaceuticals.

KI and TM are employees of Nippon Boehringer Ingelheim Co., Ltd.

IK has received honoraria from Boehringer Ingelheim, Eisai, Eli Lilly, Janssen Pharmaceutical, Meiji Seika Pharma, Mochida Pharmaceutical, Novartis Pharma, Otsuka Pharmaceutical, Shionogi, Sumitomo Pharma, Takeda Pharmaceutical, Tsumura, Viatris, and Yoshitomiyakuhin, and has received research/grant support from Asahi Kasei Pharma, Astellas, Daiichi Sankyo, Eisai, Eli Lilly, Mochida Pharmaceutical, Nihon Medi-Physics, Otsuka Pharmaceutical, Pfizer, Shionogi, Sumitomo Pharma, Takeda Pharmaceutical, and Tanabe Mitsubishi Pharma.

KN has received grants paid to his institution from Shionogi & Co., Ltd., Sumitomo Pharma Co., Ltd., Otsuka Pharmaceutical Co., Ltd., Janssen Pharmaceutical K.K., Nippon Boehringer Ingelheim Co., Ltd., and AbbVie G.K.; payment./honoraria from Sumitomo Pharma Co., Ltd., Otsuka Pharmaceutical Co., Ltd., Meiji Seika Pharma Co., Ltd., Janssen Pharmaceutical K.K., Mitsubishi Tanabe Pharma Corp., Viatris Pharmaceuticals Japan G.K., Nippon Boehringer Ingelheim Co., Ltd., Kyowa Kirin Co., Ltd., Shionogi & Co., Ltd. and Yoshitomiyakuhin Corp.; and support for transportation to attend meetings from Sumitomo Pharma Co., Ltd., Otsuka Pharmaceutical Co., Ltd., Meiji Seika Pharma Co., Ltd., Janssen Pharmaceutical K.K., Mitsubishi Tanabe Pharma Corp., Nippon Boehringer Ingelheim Co., Ltd., Shionogi & Co., Ltd., Yoshitomiyakuhin Corp. and AbbVie G.K. in the past 36 months.

## Data Availability

To ensure independent interpretation of clinical study results and enable authors to fulfil their role and obligations under the ICMJE criteria, Boehringer Ingelheim grants all external authors access to relevant clinical study data. In adherence with the Boehringer Ingelheim Policy on Transparency and Publication of Clinical Study Data, scientific and medical researchers can request access to clinical study data, typically, one year after the approval has been granted by major Regulatory Authorities or after termination of the development program. Researchers should use the https://vivli.org/ link to request access to study data and visit https://www.mystudywindow.com/msw/datasharing for further information.

## Appendix A Supplementary data

Supplementary material Image 1
