# Supplementary material for: Awareness and management of cognitive impairment associated with schizophrenia in psychiatrists and patients: Results from a cross-sectional survey
Source: Schizophr Res Cogn. 2025 Jun 27;42:100375. doi: 10.1016/j.scog.2025.100375 (PMC12269442; doi:10.1016/j.scog.2025.100375)
Supplement: Supplementary file 1 — Supplementary material [file mmc1.docx]

# Supplementary Material

# Supplementary Figure 1. Study disposition


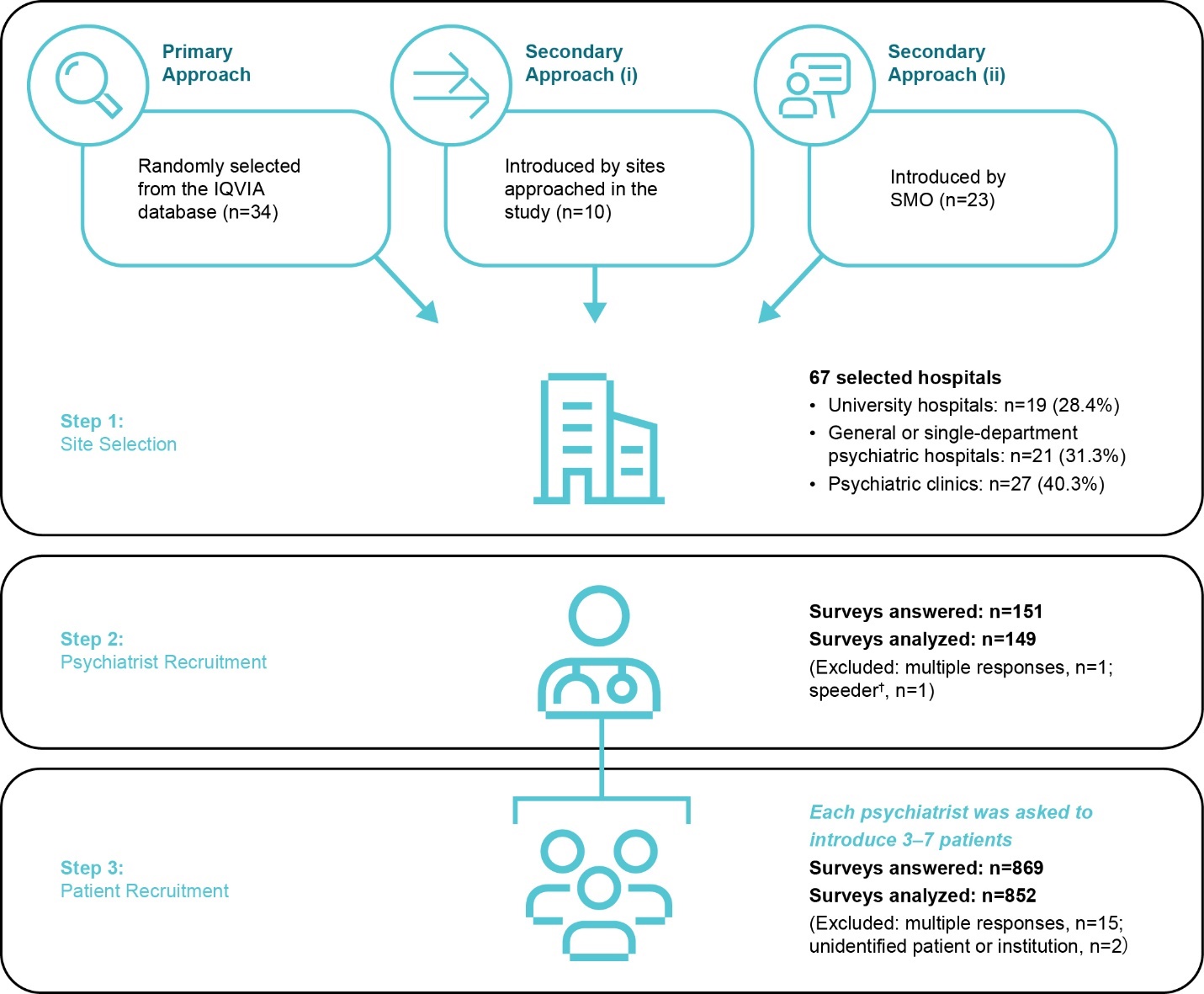


^†^A speeder was defined as a participant who completed the online questionnaire in what is perceived to be an unrealistically short period of time.

SMO, site management organizations.

# Supplementary Figure 2. Psychiatrists’ understanding of CIAS

**
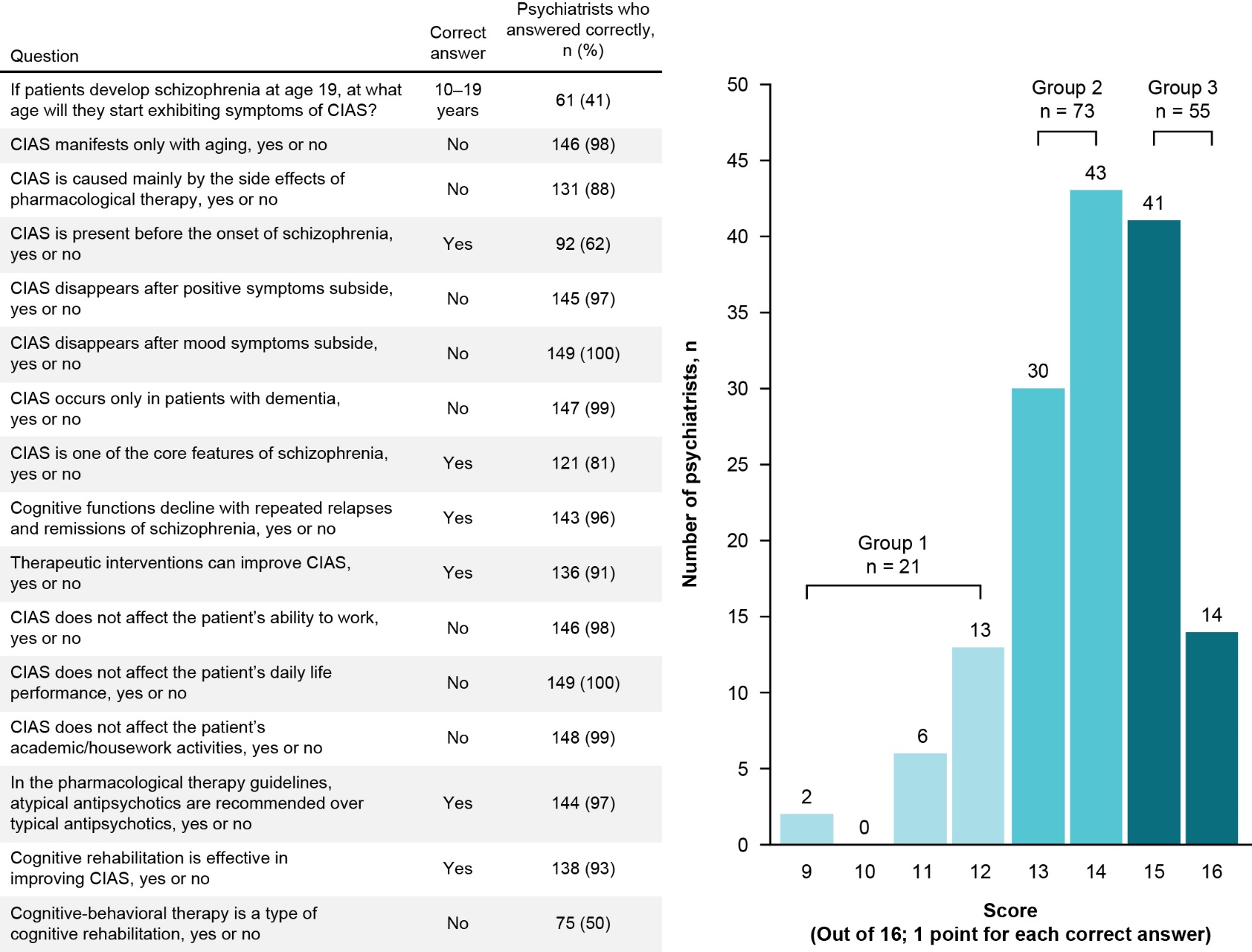
**

# Supplementary Figure 3. Proportion of inpatients and outpatients with CIAS as reported by psychiatrists, stratified by psychiatrists’ awareness of CIAS


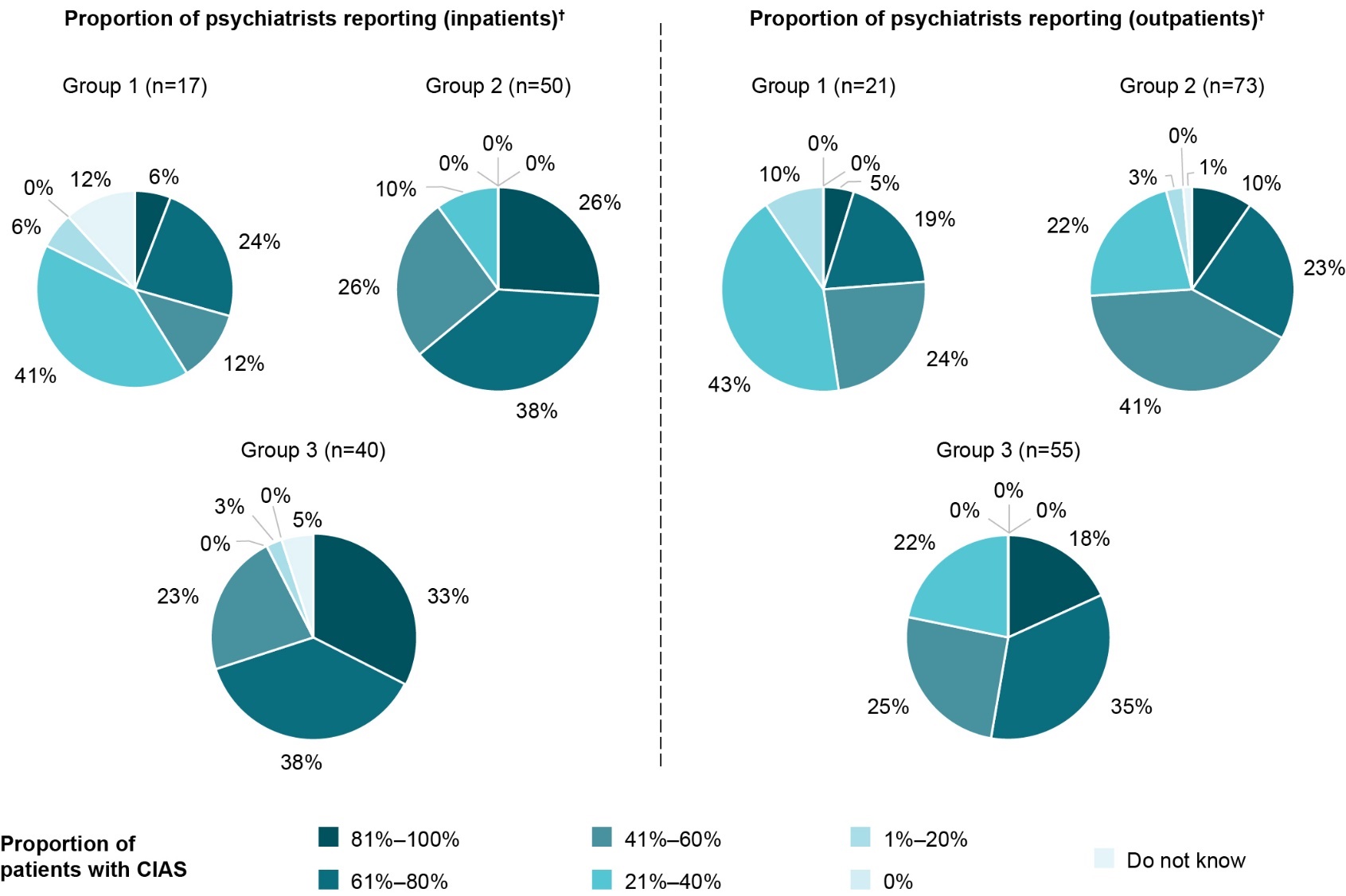


^†^Psychiatrists answered 16 questions about CIAS to assess their recognition. Psychiatrists were categorized into 3 groups based on their scores out of 16: Group 1, score 9–12 (n=21); Group 2, score 13–14 (n=73); and Group 3, score 15–16 (n=55).

# Supplementary Figure 4. Tests used by psychiatrists to evaluate CIAS in clinical practice


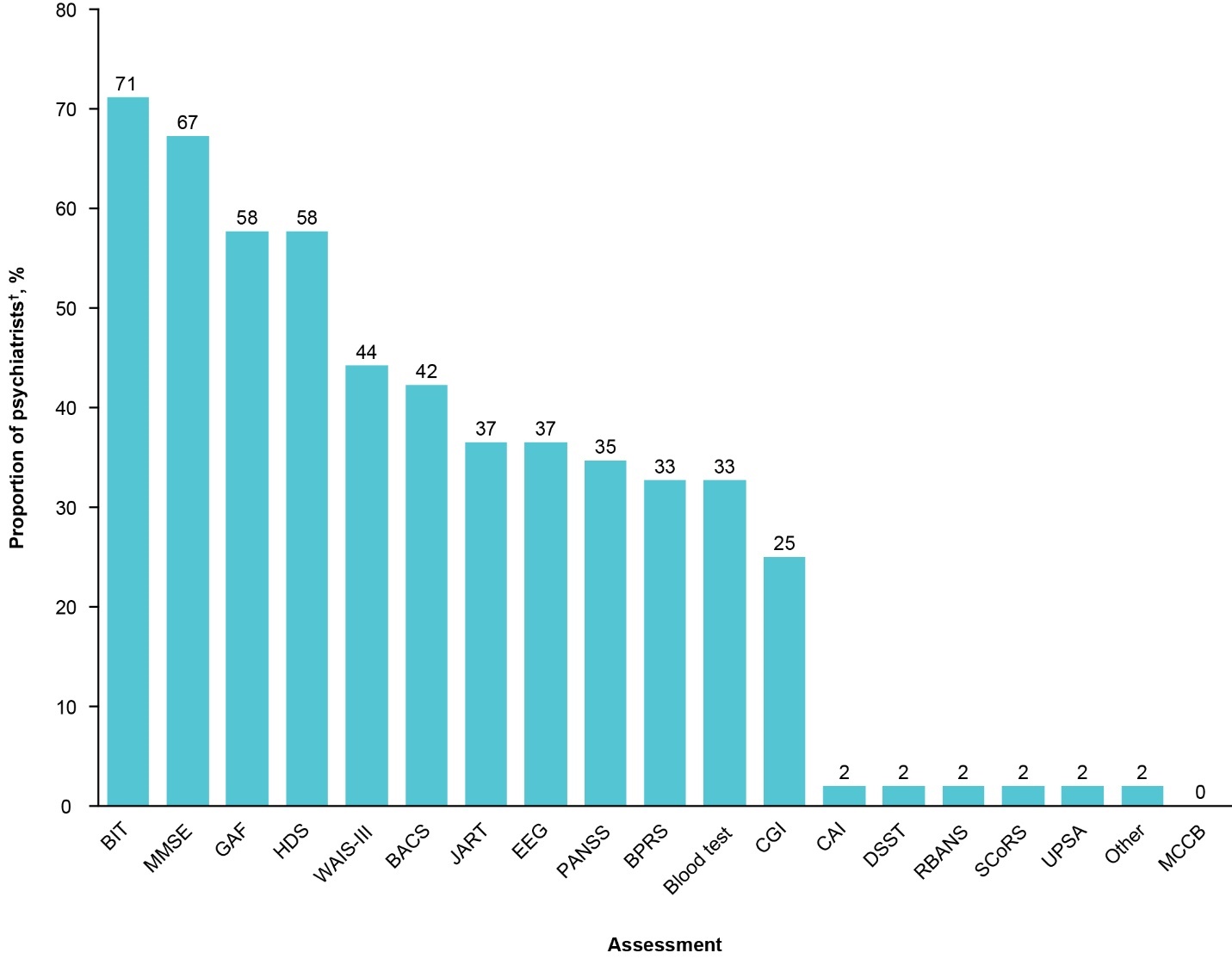


^†^Psychiatrists who use test-based assessments in an outpatient setting (n=52).

BACS, Brief Assessment of Cognition in Schizophrenia; BIT, Brain Imaging Test; BPRS, Brief Psychiatric Rating Scale; CAI, Cognitive Assessment Interview; CGI, Clinical Global Impressions; DSST, Digit Symbol Substitution Test; EEG, Electroencephalogram; GAF, Global Assessment of Functioning; HDS, Hasegawa Dementia Scale; JART, Japanese Adult Reading Test; MCCB, Matrics Consensus Cognitive Battery; MMSE, Mini Mental State Examination; PANSS, Positive and Negative Syndrome Scale; RBANS, Repeatable Battery for the Assessment of Neuropsychological Status; SCoRS, Schizophrenia Cognition Rating Scale; UPSA, UCSD Performance-based Skills Assessment; WAIS-III, Wechsler Adult Intelligence Scale 3rd Edition.

# Supplementary Figure 5. Prevalence of explanation of clinical symptoms by psychiatrists


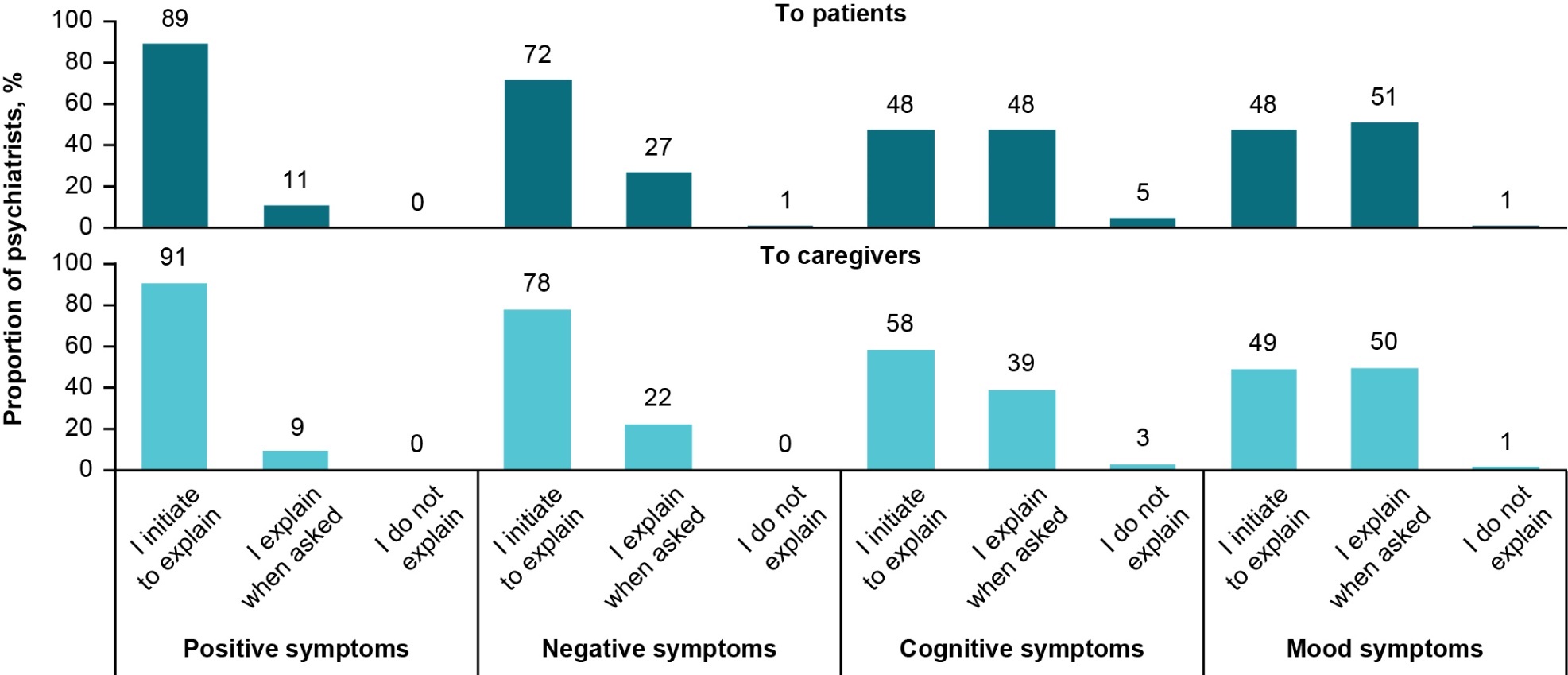


# Supplementary Figure 6. Patients’ view on achieved goals and unmet needs with current treatment^†^


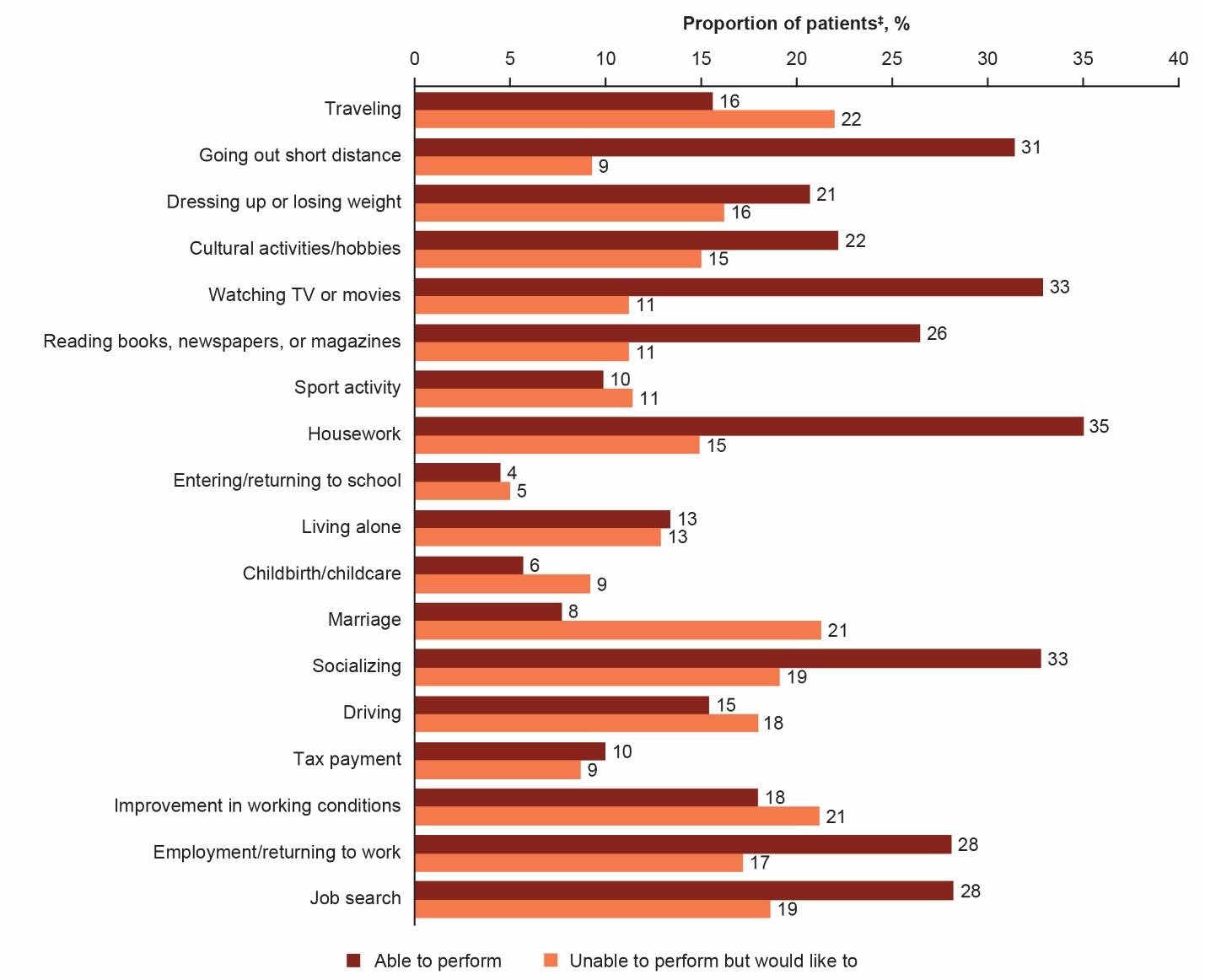


^†^Response options were: I am able to perform this task/activity; I haven't been able to perform this task/activity yet, but I would like to; None of the above. ^‡^Patients of psychiatrists who enrolled ≥3 schizophrenia patients (n=827).

# Supplementary Figure 7. Perception of patients treated by psychiatrists who responded with “I initiate to explain”


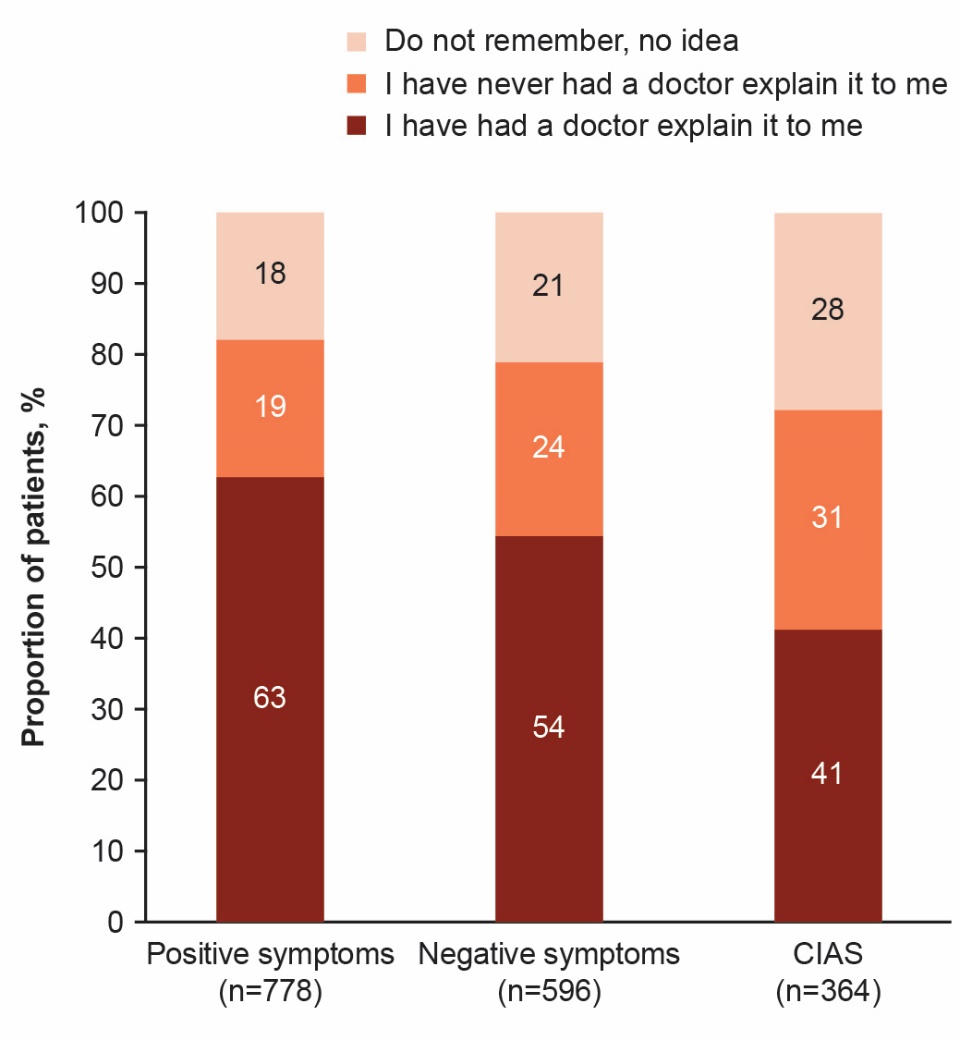


# Supplementary Figure 8. Patients’ perception of explanations of CIAS given by treating psychiatrists


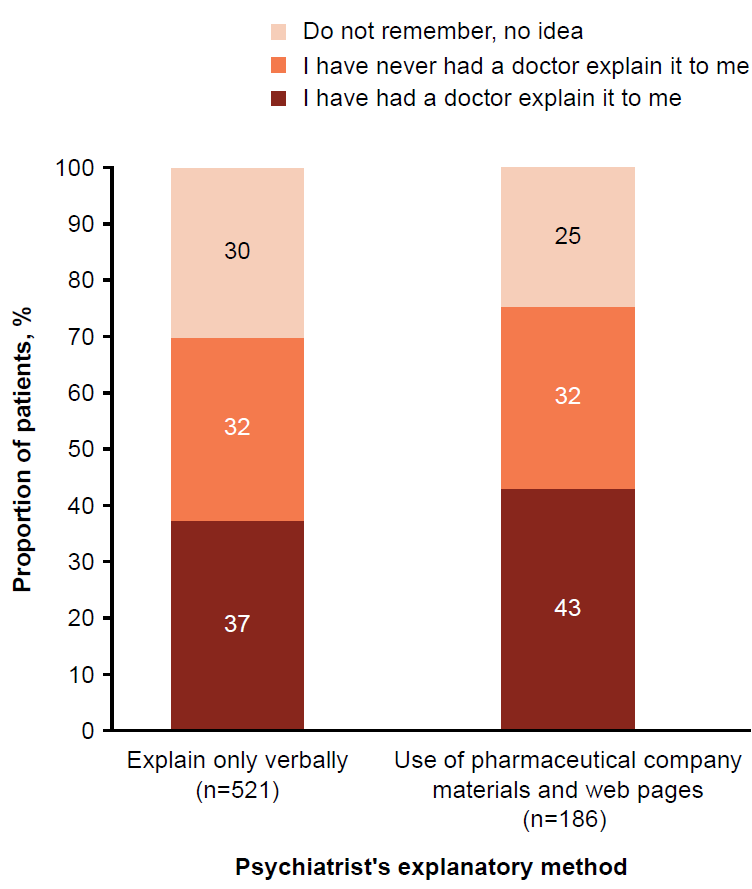


# Supplementary Appendix

## Questionnaire for psychiatrists

| **Question number** | **Base sample (respondents who answer this question)** | **Outcome** | **Questions and response options** |
| --- | --- | --- | --- |
| Q1 | All | Type of institution they work in | **Please select the type of institution you work in (if there are several, please select the place you spend the most time in a normal day for work)**  **Response options:**   - Single-department Psychiatric Hospital (Public) - Single-department Psychiatric Hospital (Private) - Department of Psychiatry in General Hospital - Department of Psychiatry in University Hospital - Psychiatry or Psychosomatic Clinic - Other |
| Q2 | All | Type of institution they work in | **Please select the main medical department you belong to.**  **Response options:**   - Psychiatry - Psychosomatic Medicine - Internal Medicine - Neurology - Neuropathic Internal Medicine - Other |
| Q3 | All | Age | **Please select your age range.**  **Response options:**   - <=29 years - 30-39 years - 40-49 years - 50-59 years - 60-69 years - >=70 years |
| Q4 | All | Qualification as a medical specialist | **Please select all of the following that apply to you for certified medical qualifications. (Select all that apply)**   - Designated Mental Health Physician - Certified Psychiatrist of the Japanese Society of Psychiatry and Neurology - Japanese Society of Psychiatry and Neurology Psychiatry Preceptor - Board Certified Clinical Neuropsychopharmacologist - Japan Medical Association Certified Occupational Physician - Dementia support doctor - CPMS (Clozaril Patient Monitoring Service) Registered Physician - Other - Not applicable   **Response options:**   - I had it in the past but I do not have it now - I have it now - I plan to receive certification within the next two years |
| Q5 | All | Experience of clinical research and/or clinical trials related to schizophrenia | **Please select any of the following that you have participated related to schizophrenia (Select all that apply)**  **Response options:**   - Clinical research - Company-initiated clinical trials/physician-initiated clinical trials - Preparation of drug treatment guidelines for schizophrenia - Generation of treatment algorithm - Participation in conferences (at least once a year) - Participation in local seminars (at least once a year) - Participation in seminars on drug treatment guidelines and algorithms for schizophrenia - Other - Not applicable |
| Q6 | All | Experience treating schizophrenia (number of patients) | **Please select the number of patients you have treated for the following conditions in the past 3 months.**  ***Please answer the total number of patients treated in clinical practice, excluding those involved only in clinical trials. A ballpark number is sufficient.**   - Schizophrenia - Depression - Bipolar disorder   **Response options:**   - None - 1-30 patients - 31-60 patients - 61-99 patients - 100 or more patients |
| Q7 | Psychiatrists who chose ‘Schizophrenia’ in Q6 | Experience treating schizophrenia (number of hospitalized patients) | **Please select the number of hospitalized patients with schizophrenia you have treated in the past three months. *A ballpark number is sufficient.**  **Response options:**   - Do not have in-patient facilities - None - 1-30 patients - 31-60 patients - 61-99 patients - 100 or more patients |
| Q8 | Psychiatrists who chose ‘Schizophrenia’ in Q6 | Experience treating schizophrenia (age range of patients treated) | **Please tell us the age ratio of schizophrenic patients you have treated in the past three months. *Please make sure the numbers add up to 100%. *A ballpark number is sufficient.**  **Response options:**   - <=29 years _% - 30-59 years _% - >= 60 years _% |
| Q9 | All | Experience treating schizophrenia (stages of schizophrenia treated) | **Among your patients, which phase of schizophrenia do you see the most? *Please make sure the numbers add up to 100%. A ballpark number is sufficient.**  **Response options:**   - Acute phase _% - Maintenance/stable phase _% |
| Q10 and Q11 | All | The extent of understanding and referring to the Guideline for Pharmacological Therapy of Schizophrenia 2022 (published by The Japanese Society of Neuropsychopharmacology) | **How familiar are you with the content of the Guideline for Pharmacological Therapy of Schizophrenia 2022? Please answer on a scale of 0 to 5, with 0 being the lowest (not familiar at all) and 5 being the highest (very familiar).**  **Response options:**   - 0 (Not familiar at all),1,2,3,4,5 (Very familiar with the content)   **When treating schizophrenia, to what extent do you refer to the contents of the Guideline for Pharmacological Therapy of Schizophrenia 2022 in your actual treatment? Please answer on a scale of 0 to 5, with 0 being the lowest (no reference at all) and 5 being the highest (often refer to).**  **Response options:**   - 0 (No reference at all),1,2,3,4,5 (Often refer to) |
| Q12 | All | Treatment priorities for acute phase / maintenance phase | **Please select the following items you prioritize upon treating patients with schizophrenia in the [acute phase] and [maintenance phase/stable phase], respectively. (Select up to three choices each)**   - Acute phase - Maintenance/stable phase   **Response options:**   - Controlling positive symptoms - Reducing side effects - Improving social functioning - Improving negative symptoms - Improving depressive symptoms - Improving cognitive impairment - Minimizing sedative use - Improving interpersonal skills - Improving daily life performance - Other - None of the above |
| Q13 | All | Patient’s needs and expectations of treatment outcomes with antipsychotics in the acute phase and maintenance phase | **Please select up to three symptoms that "patients" most commonly expect to improve with drug treatment in the [acute phase] and [maintenance phase], respectively.**   - Acute phase - Maintenance phase   **Response options:**   - Hallucinations - Delusion - Recurrence/Relapse - Emotional Dullness - Social Apathy - Concentration Difficulty/Attention Disturbance - Memory Impairment - Depressive Symptoms - Anxiety Symptoms - Sleeping Disorder - Impulsivity Control - Other - None of the above |
| Q14 | All | Clinical symptoms to be treated for schizophrenic patients to reintegrate into society | **Which clinical symptom(s) associated with schizophrenia is particularly important, in your opinion, to be treated for patients with schizophrenia to reintegrate into society? * Please answer within the realm of the patient's clinical symptoms, excluding factors such as support from others and social engagement.**  ***If more than one applies, please select up to three items that you consider more important than others.**  **Response options:**   - Positive symptoms - Impulsivity - Negative symptoms - Depressive symptoms - Anxiety symptoms - Cognitive impairment - Sleep disorder - Suicidal ideation - Other - None of the above |
| Q15 | All | Psychiatrists’ perspectives on the cause of CIAS in schizophrenic patients | **Please select what you think are the causes of CIAS. (Select all that apply) *CIAS refers to clinical symptoms with reduced performance skills, attention and memory.**  **Response options:**   - Positive symptoms - Negative symptoms - Characteristics of schizophrenia - Depressive symptoms - Recurrence/relapse - Aging - Side effects of drug therapy - Long-term hospitalization - Social withdrawal/reclusiveness - Genetic factors - Dysfunction of neurotransmission - Other - None of the above |
| Q16 | All | Psychiatrists’ perspectives on the age range of schizophrenic patients presenting CIAS | **Assuming a patient develops schizophrenia at the age of 19, at what age do you think he or she will develop cognitive impairment?**  ***CIAS refers to clinical symptoms of impaired executive function, attention, memory, and so on.**  **Response options:**   - Teen - 20s - 30s - 40s - 50s |
| Q17 | All | Psychiatrist’s views on the recognition of CIAS | **If you receive complaints from a schizophrenic patient in their 20s or 30s or a family member of the patient about the following issues, please answer which symptom of schizophrenia you believe is the cause.**   - Failing to follow instructions from others, making mistakes, and/or unable to remember - Inability to plan trips or visits, and stop travelling long distances - Inability to go to the bathroom - Inability to dress properly and to perform self-care - Inability to manage and take medication properly - Taking more time to do household tasks that used to be done timely - Inability to concentrate on reading, watching TV or movies - Inability to memorize the date, days of the week, frequented places, or well-known people - Losing interest and motivation, cease participation in hobbies and activities   **Response options:**   - Positive symptoms - Negative symptoms - CIAS - None of the above (suspect other conditions aside from schizophrenia) |
| Q18 | All | Understanding of CIAS and differentiation from dementia and/or cognitive decline by aging | **Please read the following descriptions of CIAS and select 〇 if correct and × if incorrect**   - CIAS manifests only with aging - CIAS is caused mainly by the side effects of pharmacological therapy - CIAS is present before the onset of schizophrenia - CIAS disappears after positive symptoms subside - CIAS disappears after mood symptoms subside - CIAS occurs only in patients with dementia - CIAS is one of the core features of schizophrenia   **Response options:**   - **×** - 〇 |
| Q19 | All | Perception of CIAS in clinical practice | **Please read the following descriptions of CIAS and select 〇 if correct and × if incorrect**  *** The questions do not necessarily have answers; some will ask for your opinion.**  ***There are four pages of questions all together**  *** Please understand that some questions may be redundant**  *** Please choose the closest answer based on your opinion if you are unsure of or have difficulty choosing an answer**   - Cognitive functions decline with repeated relapses and remissions of schizophrenia - Therapeutic interventions can improve CIAS - Improving CIAS is expected to rejoin society and promote discharge from long-term hospitalization   **Response options:**   - **×** - 〇 |
| Q20 | All | Perception of functional outcome of CIAS | **Please read the following descriptions of CIAS and select 〇 if correct and × if incorrect**  *** The questions do not necessarily have answers; some will ask for your opinion.**  *** There are four pages of questions all together**  *** Please understand that some questions may be redundant**  *** Please choose the closest answer based on your opinion if you are unsure of or have difficulty choosing an answer**   - CIAS does not affect patients’ ability to work - CIAS does not affect patients’ daily life performance - CIAS does not affect patients’ academic/housework activities   **Response options:**   - **×** - 〇 |
| Q21 | All | Perception of CIAS in clinical practice | **Please read the following descriptions of CIAS and select 〇 if correct and × if incorrect**  *** The questions do not necessarily have answers; some will ask for your opinion.**  *** There are four pages of questions all together**  *** Please understand that some questions may be redundant**  *** Please choose the closest answer based on your opinion if you are unsure of or have difficulty choosing an answer**   - In the pharmacological therapy guidelines, atypical antipsychotics are recommended over typical antipsychotics - Cognitive rehabilitation is effective in improving Cognitive impairment associated with schizophrenia - Cognitive-behavioral therapy (CBT) is a type of cognitive rehabilitation   **Response options:**   - **×** - 〇 |
| Q22 | All | Percentage of patients presenting CIAS | **Please answer the percentage of patients with CIAS, in your opinion, among your patients.**   - Outpatient - Inpatient   **Response options:**   - I do not know - 0%（No patient presents with cognitive impairment - 1-20% - 21-40% - 41-60% - 61-80% - 81-100% |
| Q23 | All | Psychiatrist’s perception of CIAS in treatment goals | **When setting treatment goals as part of the treatment management for schizophrenic patients, do you take into account the patients’ social functions and living standards prior to the onset of symptoms?**  **Response options:**   - Yes - No - I do not know |
| Q24 | Psychiatrists who chose options other than ‘I do not know’ or ‘0%’ in Q22 | Percentage of patients who aim to improve cognitive impairment | **What percentage of schizophrenic patients whom you treat aim to improve their cognitive impairment?**  **Response options:**   - 0% - 1-20% - 21-40% - 41-60% - 61-80% - 81-100% |
| Q25 | Psychiatrists who chose options other than ‘0%’ in Q24 | Perception of CIAS in clinical practice | **What kind of treatment do you provide for CIAS? Please select all that apply.**  **Response options:**   - Reduction of antipsychotic dosage - Prescription of dementia medication - Reduction of anticholinergic medication and benzodiazepine receptor agonists - Selection of antipsychotic drugs considering effects on cognitive impairment - Adult day care services - Cognitive rehabilitation - Social skills training - Repetitive transcranial magnetic stimulation - Transcranial direct current stimulation - Other |
| Q26 | All | History of CIAS assessment in clinical practice | **Do you have experience evaluating CIAS in the daily clinical practice?**  **Response options:**   - No, we do not perform CIAS evaluation - Yes, we perform CIAS evaluation (assessed by consultation, or neuropsychological tests, etc.) |
| Q27 | Psychiatrists who chose ‘Yes’ in Q26 | Perceptions on the assessment of CIAS in clinical practice | **How do you perform CIAS evaluation? (Select all that apply)**  **Response options:**   - Evaluation by interview - Evaluation by tool (neuropsychological tests, etc.) - Other |
| Q28 | Psychiatrists who chose ‘Evaluation by tool’ in Q27 | Percentage of patients assessed for CIAS in clinical practice | **What percentage of patients with schizophrenia are evaluated for cognitive impairment by neuropsychological testing?**   - Outpatient - Inpatient (Psychiatrists who chose ’inpatient‘ in Q7)   **Response options:**   - Evaluation not performed - Some patients - Half of the patients - All patients |
| Q29 | Psychiatrists who chose ‘No, we do not perform CIAS evaluation’ in Q26 | Perception on the assessment of CIAS in clinical practice (reasons for not assessing CIAS) | **Please select all the appropriate reasons for not assessing CIAS from the options below.**  **Response options:**   - Assessment of CIAS has no particular benefit to the patient for treatment management - Assessment of CIAS has no particular benefit for the functional recovery of patients - Patients have not reached the age range to suspect cognitive impairment - Assessment of CIAS will impose burden on the patients - Assessment of CIAS is unclear - Treatment and intervention following evaluation is unclear; do not know what to do with results - Neuropsychological tests are not covered by health insurance - Lack of resources to perform neuropsychological tests - Lack of time to do the neuropsychological tests - Lack of space for neuropsychological testing (e.g., private room or computer terminal for testing) - Other |
| Q30 | Psychiatrists who chose ‘Evaluation by tool’ in Q27 | Types of tests to assess CIAS in clinical practice | **Please select an evaluation test(s) that is performed in your practice to evaluate CIAS (Select all that apply).**  **Response options:**   - BACS (The Brief Assessment of Cognition in Schizophrenia) - BPRS (Brief Psychiatric Rating Scale) - CAI (The Cognitive Assessment Interview) - CGI (Clinical Global Impressions) - DSST (The Digit Symbol Substitution Test) - GAF (Global Assessment of Functioning) - JART (Japanese Adult Reading Test) - MCCB (MATRICS Consensus Cognitive Battery) - MMSE (Mini Mental State Examination) - PANSS (Positive and Negative Syndrome Scale) - RBANS (Repeatable Battery for the Assessment of Neuropsychological Status) - SCoRS (The Schizophrenia Cognition Rating Scale) - UPSA (UCSD Performance-based skills Assessment) - WAIS-III (Weschler Adult Intelligence Scale 3rd edition) - Blood test - Brain imaging test - Electroencephalogram (EGG) - Hasegawa dementia scale - Other |
| Q31 | Psychiatrists who chose ‘Yes, we perform CIAS evaluation (assessed by consultation, or neuropsychological tests, etc.)’ in Q26 | Perception on the assessment of CIAS in clinical practice (reason to assess CIAS) | **Please tell us what you consider as the trigger(s) for assessing CIAS in your practice. (Select all that apply)**  **Response options:**   - I considered the effects of cognitive impairment from the patient's symptoms - Nurses and co-medicals told me about the effects of cognitive impairment on patients - Patients complained about cognitive impairment - The patient told me that he wanted to work or return to school - The patient told me that he wanted to get married - The patient told me that he wanted to live alone - The patient’s family complained about problems related to cognitive impairment - I had the opportunity to evaluate CIAS through participation in clinical research - I had the opportunity to evaluate CIAS through participation in a clinical trial - The patient started to show increased forgetfulness - The patient has become more senior in age - I learned the importance of evaluating cognitive function from academic conferences or papers - Other |
| Q32 | Psychiatrists who chose answers other than ‘Do not have in-patient facilities’ or ‘None’ in Q7 | Percentage of schizophrenia inpatients aware of their cognitive impairment | **Among the schizophrenic in-patients you treat, what percentage do you feel are aware of their own cognitive impairment? *Please make sure the numbers add up to 100%. *A ballpark number is sufficient.**  **Response options:**   - Aware _% - Not aware _% |
| Q33 | All | Percentage of schizophrenia outpatients aware of their cognitive impairment | **Among the schizophrenia out-patients you treat, what percentage do you feel are aware of their own cognitive impairment? *Please make sure the numbers add up to 100%. *A ballpark number is sufficient.**  **Response options:**   - Aware _% - Not aware _% |
| Q34 | All | Perception of burdens felt by caregivers of schizophrenic patients | **Please select any "problems" that are raised by family members or cohabitants of schizophrenic patients.**  **Response options:**   - Unable to live with the patient at home - Cannot let the patient live alone - Patient is unaware of the disease - Even though the symptoms have subsided, the patient has a hard time reintegrating into society - I don't know how to engage with the patient, my relationship with the patient has deteriorated due to the illness - The patient does not take his/her medication - The patient dislikes going to the hospital - I feel burdened caring for and supporting the patient for his/her physical illnesses caused by side effects - Lack of support to consult about troubles - Discrimination and prejudice, lack of understanding from others - Caregiver's own poor health makes it difficult to support the patient - Lack of secondary caregiver - None - Other |
| Q35 | All | Explaining symptoms of schizophrenia to patients and caregivers | **How do you explain the following symptoms pertaining to schizophrenia to “patients” and to “families or cohabitants of patients”? Please select the answer that applies most.**  **To whom**   - Patients - Families and cohabitants of patients   **Symptoms you explain**   - Positive symptoms - Negative symptoms - Cognitive impairment - Mood symptoms   **Response options:**   - I initiate to explain - I explain when asked - I do not explain |
| Q36 | Psychiatrists who select ‘I initiate to explain’ or ‘I explain when asked’ in Q35 | Explaining cognitive impairment to patients with schizophrenia | **Please select the appropriate timing for explaining "cognitive impairment" to patients with schizophrenia from the following (Select all that apply)**  **Response options:**   - During hospitalization - During outpatient care - During institutional disease education/psychoeducational program - Other |
| Q37 | Psychiatrists who answered Q36 | Explaining cognitive impairment to patients with schizophrenia | **Please answer each of the following timings for explaining the cognitive impairment to patients with schizophrenia.**   - During hospitalization - During outpatient care - During institutional disease education/psychoeducational program - Other   **Response options:**   - I explain to a part of the patients - I explain to about half of the patients - I explain to almost all of the patients |
| Q38 | Psychiatrists who select ‘I initiate to explain’ or ‘I explain when asked’ in Q35 | Explaining cognitive impairment to patients with schizophrenia | **Please answer what methods you use to explain cognitive impairment to patients with schizophrenia. (Select all that apply)**  **Response options:**   - Explain verbally only - Explain using web contents and other materials created by universities and medical institutions - Explain using web contents and handouts created by academic societies, research groups, etc. - Explain using content and materials from a treatment guideline - Explain using web contents and handouts created by pharmaceutical companies - Other |
| Q39 | All | Intention to join Research Step 2 | **Would you be willing to participate in a subsequent survey (i.e., Research Step 2)? In Research Step 2, you will be asked to subjectively assess the level of cognitive impairment (e.g., NCI/mild/moderate/severe) of patients who participated in the first step of the survey and consented to join the second step.**   - **You will be contacted with contract details if you agree to participate in the Research Step 2** - **You will be compensated for your time if you participate in Research Step 2** - **Patients are required to take a cognitive ability test and a simple self-evaluation in Research Step 2**   **Response options:**   - Yes, I am willing to participate in Research Step 2. - No, I do not wish to participate in Research Step 2. |

## Questionnaire for patients

| Q1 | All | Gender | **Please select your gender.**  **Response options:**   - Male - Female - Neither - Do not want to answer |
| --- | --- | --- | --- |
| Q2 | All | Age | **Please select your age range.**  **Response options:**   - <=19 years - 20-29 years - 30-39 years - 40-49 years - 50-59 years - >=60 years |
| Q3 | All | Living status/environment | **Please select the item that best describes your current living environment.**  **Response options:**   - Living alone - Living with spouse or partner - Living with family other than spouse or partner - Living in group home or care home* - Hospitalized - Other - * Group home/Care home: Services for mentally disabled persons who are anxious about living alone to live together and receive counseling on daily living and support for household chores. |
| Q4 | All | Occupation | **Please select the closest answer in regard to your current employment status and/or use of employment services**  **Response options:**   - Full-time employee (regular employment) - Full-time employee (employment of persons with disabilities) - Contract/temporary employee - Self-employed/sole proprietor - Part-time job - Type A workplace (continued employment support with employment contract and salary) - Type B workplace (continued employment support where there is no employment contract and work wages are paid) - Employment transition support (service for receiving training for regular employment) - Housemaker - Student - Other - None of the above |
| Q5 | All | Utilization of social resources and services | **Please choose the service(s) you currently use for schizophrenia. (Select all that apply)**  **Response options:**   - Home care services - Short stay - Day care/Night care/Short care - Community activity support center - Other - No services currently in use   *Home care services: Services that provide daily living assistance in the home.  *Short stay: A service that allows people who live with their families to stay in a place other than their homes for a short period of time.  *Day care/Night care/Short care: day care facilities that provide various group activities for the purpose of social participation and social reintegration.  *Community activity support center: Facilities where users can talk and work with other users and consult with staff about problems in daily life in preparation for social reintegration. |
| Q6 | All | Experience of schizophrenia symptoms | **Since the onset of schizophrenia, have you ever experienced any of the following symptoms?**   - Positive symptoms (e.g., delusions, hallucinations (auditory hallucinations)) - Negative symptoms (e.g., lack of motivation, lack of facial expression) - Cognitive impairment (e.g., inability to concentrate, inability to remember, inability to remember things, inability to arrange things) - Mood symptoms (e.g., depression, anxiety, addiction)   **Response options:**   - Previously experienced - Currently experiencing - Never experienced |
| Q7 | All | Current treatment for schizophrenia (pharmacological) | **Please select the medical treatment(s) you currently use for schizophrenia. (Select all that apply)**  **Response options:**   - Oral medicine - Injection - Tape treatment - Have never received medical treatment - Previously treated with medication but not now - Other - I do not know |
| Q8 | All | Current treatment for schizophrenia (non-pharmacological) | **Have you ever received the following treatment (non-drug treatment) for schizophrenia? (Select all that apply)**   - Cognitive rehabilitation (cognitive remediation therapy, cognitive corrective therapy) - Social skills training (SST) - Cognitive behavioral therapy (CBT) - Transcranial magnetic stimulation therapy (TMS) - Electroconvulsive therapy - Never received - I do not know   *Cognitive rehabilitation: Non-pharmacological therapy aimed at restoring cognitive function. Typical examples of cognitive rehabilitation for schizophrenia patients include NEAR and VCAT-J.  *Social skills training: a type of psychiatric rehabilitation related to interpersonal relationships and self-management.  *Cognitive behavioral therapy: a type of psychotherapy that promotes problem solving by working on the way people think and perceive things (cognition).  *Transcranial magnetic stimulation therapy: A non-invasive treatment in which a magnetic field generator (coil) is attached over the head to deliver magnetic pulse stimulation. |
| Q9 | All | Perceived burdens from schizophrenia | **Please select the most appropriate answer on the degree of burden you currently experience for the following statements because of schizophrenia.**   - Lack of confidence in oneself - Have false assumptions or auditory hallucinations - Depressive symptoms - Alcohol or tobacco addiction - Feel rejected by others - Feel discriminated against or prejudice - Feel anxiety and loneliness when thinking about the future and relationships - Unable to commit to one job   **Response options:**   - No burden - Some burden - Heavy burden |
| Q10 | All | Perceived burdens from schizophrenia | **Please select the most appropriate answer on the degree of burden you currently experience for the following statements because of schizophrenia.**   - Unable to read newspapers, books, or magazines or follow plots of TV shows or movies - Unable to remember things just told to me or instructions from others - Have difficulty remembering what I want to say or expressing my thoughts smoothly - Get confused during conversations or have difficulty in understanding what others say - Unable or perform tasks I could do before (e.g., housework, study, or work) or they take longer   **Response options:**   - No burden - Some burden - Heavy burden |
| Q11 | All | Perceived burdens from schizophrenia | **Please select the most appropriate answer on the degree of burden you currently experience for the following statements because of schizophrenia.**   - Unable to manage a sudden change in schedule - Unable to maintain concentration - Unable to remember where I put things - Unable to remember instructions or new information without taking notes   **Response options:**   - No burden - Some burden - Heavy burden |
| Q12 | All | Perceived burdens from schizophrenia | **Please select the most appropriate answer on the degree of burden you currently experience for the following statements because of schizophrenia.**   - Continuation to take medication - Side effects of drugs (weight gain, difficulty in moving the body, etc.) - Forgetfulness to take medication - Physical symptoms such as fatigue and drowsiness - Taking multiple medications   **Response options:**   - No burden - Some burden - Heavy burden |
| Q13 | All | Duration of the relationship with current treating physician | **How many years have you been seeing your current doctor?**  **Response options:**   - Less than one year - More than one year but less than 3 years - More than 3 years but less than 5 years - More than 5 years but less than 10 years - 10 years or more |
| Q14 | All | Explanation from physician about each clinical symptom of schizophrenia including CIAS | **Has your doctor ever explained to you about the following symptoms related to schizophrenia? Please select the one that applies to you.**   - Positive symptoms (e.g., delusions, hallucinations (auditory hallucinations)) - Negative symptoms (e.g., lack of motivation, lack of facial expression) - Cognitive impairment (e.g., inability to concentrate, inability to remember, inability to remember things, inability to arrange things) - Mood symptoms (e.g., depression, anxiety, addiction)   **Response options:**   - I have had a doctor explain it to me - I have never had a doctor explain it to me - Do not remember, no idea |
| Q15 | All | Topics patients wish to discuss with their physicians but have not been able to | **Do you feel that there are “topics you can discuss with your physician” or “topics you would like to discuss with your physician but have not been able to” during your regular medical examinations? Please choose the topic(s) that best applies to you. (Select all that apply).**   - About symptoms or physical disorders - About problems in daily life - About work, housework, or schoolwork - About your final situation - About the effect of medicine - About your interest in a new medicine - About side effects of medicine - About the content of treatment - About treatment goals - Other - None of the above   **Response options:**   - I can discuss this topic with my physician - I have not been able to discuss with the physician, but I would like to |
| Q16 | All | Average and expected amount of time for physician consultation | **How long does a normal consultation with your physician take? How long would you expect it to last for? Please select the answer that best applies to each of the following.**   - Average duration of time for doctor’s visits - Amount of time you expect your physician will spend   **Response options:**   - Less than 5 minutes - More than 5 minutes, but less than 10 minutes - More than 10 minutes, but less than 30 minutes - 30 minutes or more |
| Q17 | All | Other stakeholders to consult on disease and treatment goals | **Have you ever consulted or been explained about the symptoms of schizophrenia from anyone other than a physician? (Select all that apply)**  **Response options:**   - Nurse - Psychologist (certified psychologist, clinical psychologist, counselor) - Occupational therapist - Pharmacist - Social worker in mental health - Peer supporter - Public health nurse - Care manager - Registered nutritionist - Physical therapist - Clinical laboratory technician - Home helper - Reception clerk at a hospital or clinic - Employment transition support service destinations and staff at workshops - Have never consulted or never been explained |
| Q18 | All | Experience and ways of searching information on schizophrenia | **Have you ever searched for information about the following issues?**   - Positive symptoms (e.g., delusions, hallucinations (auditory hallucinations)) - Negative symptoms (e.g., lack of motivation, lack of facial expression) - Cognitive impairment (e.g., inability to concentrate, inability to remember, inability to remember things, inability to arrange things) - Mood symptoms (e.g., depression, anxiety, addiction)   **Response options:**   - I have done some research previously - Never |
| Q19 | The one selected ‘I have done some research’ in Q18 | Experience and ways of searching information on schizophrenia | **Please select the item you use when looking up information about schizophrenia.**  **Response options:**   - Internet searches (e.g., Google, Yahoo, etc.) - Video sharing site (e.g., YouTube, etc.) - Social Media - Blog - Pharmaceutical company web page about schizophrenia - Books (all reading material, including medical books, comic books, etc.) - Posters and brochures in hospitals and clinics (disease education, psychoeducation programs, etc.) - Seminars or study sessions sponsored by hospitals and clinics - Patient groups - Family groups - Self-help group/peer supporter - Academic conferences (academic events in which physicians participate) - Public lectures - Other |
| Q20 | All | Achieved goals, satisfaction, and unmet needs with current treatment | **Please select activities or tasks that i) you are able to perform because you have been treated for schizophrenia; and ii) you cannot presently perform but would like to in the future. (Select all that apply)**   - Job search - Employment/Returning to work - Improvement in working conditions - Tax payment - Driving - Socializing - Marriage - Childbirth/Childcare - Living alone - Entering/Returning to school - Housework - Sport activity - Reading books, newspapers or magazines - Watching TV or movies - Cultural activities/Hobbies - Dressing up or losing weight - Going out short distance - Traveling - Other - None of the above   **Response options:**   - I am able to perform this task/activity - I haven't been able to perform this task/activity yet, but I would like to |
| Q21 | Patients who selected other than ‘none of the above’ in Q20 | Topics patients wish to discuss with their physicians | **Have you ever discussed your problems and expectations on (*items from previous question shown here*) from the previous question with your physician?**  **Response options:**   - Yes - No |
| Q22 | Patients who selected ‘No’ in Q21 | The reasons why patients cannot discuss problems and expectations with physicians | **What are the reasons that you couldn’t discuss with your physician regarding tasks and activities you have not been able to perform but would like to? (Select all that apply)**  **Response options:**   - There is not enough time for discussion - It is difficult for me to express my expectations - I cannot organize my thoughts well - I never thought of having a discussion - Other - No reason |
| Q23 | All | Motivation and expectations to maintain medication adherence | **Please select all that apply on what improvements you expect from treatment with medications (Select all that apply).**  **Response options:**   - Prevention of recurrence/relapse (To prevent symptoms of previous illness from appearing) - Reduction of hallucinations (e.g., Auditory Hallucinations) - Reduction of delusions - Emotional numbing (Easier to express emotions) - Social indifference, withdrawal (Getting out of the house) - Difficulty in concentrating, attention disorder (Can pay attention to things and concentrate) - Memory impairment (Can remember things that happened immediately before) - Depressive symptoms (Patient begins to feel pleasure) - Anxiety symptoms (Become to feel less anxious) - Sleep disorder (Sleeping well) - Impulse control (Become able to suppress impulsive feelings) - None |
| Q24 | Patients who selected answers other than ‘Have never received medicine treatment’ in Q7 | Motivation and expectations to maintain medication adherence | **Please select the following that apply to you as the reason why you continue to take your medication (Select all that apply).**  **Response options:**   - I can feel the therapeutic effect - The drug has fewer side effects - I have clear goals for taking the medication - I receive explanation and support from my physician - I receive support from health professionals other than physicians - I receive support from families or friends - Other - No reason |
| Q25 | All | Willingness to join 2^nd^ step | **Would you be willing to join subsequent research that involves taking a cognitive ability test (i.e., similar to a computer game that measures the overall ability on memory, judgment and concentration) and a short survey under the following conditions?**   - **The research will take a maximum of two hours. You will be reimbursed for your time.** - **Test results will be anonymized and processed; in this regard, please note that you will not be able to see your results.** - **Any personal information obtained will be deidentified prior to sharing with a third party.** - **Response options:** - Yes, I am willing to join - No, please count me out |
| Q26 | All |  | **Finally, please answer the approximate time it took for you to answer this questionnaire.**  **Response options:**   - Less than 15 minutes - 16-30 minutes - 31-60 minutes - 61 minutes or more |
